# Macro-, Micro- and Nanomechanical Characterization of Crosslinked Polymers with Very Broad Range of Mechanical Properties

**DOI:** 10.3390/polym12122951

**Published:** 2020-12-10

**Authors:** Miroslav Slouf, Beata Strachota, Adam Strachota, Veronika Gajdosova, Vendulka Bertschova, Jiri Nohava

**Affiliations:** 1 Institute of Macromolecular Chemistry, Czech Academy of Sciences, Heyrovskeho nam. 2, 16206 Prague 6, Czech Republic; beata@imc.cas.cz (B.S.); strachota@imc.cas.cz (A.S.); gajdosova@imc.cas.cz (V.G.); 2 Anton Paar TriTec SA, Vernets 6, 2035 Corcelles, Switzerland; vendulka.bertschova@tescan.com (V.B.); jiri.nohava@anton-paar.com (J.N.)

**Keywords:** microindentation, nanoindentation, depth-sensing indentation, crosslinked polymers, stiff vitrified networks, soft elastic rubbers, glass transition temperature

## Abstract

This work is focused on the comparison of macro-, micro- and nanomechanical properties of a series of eleven highly homogeneous and chemically very similar polymer networks, consisting of diglycidyl ether of bisphenol A cured with diamine terminated polypropylene oxide. The main objective was to correlate the mechanical properties at multiple length scales, while using very well-defined polymeric materials. By means of synthesis parameters, the glass transition temperature (*T*_g_) of the polymer networks was deliberately varied in a broad range and, as a result, the samples changed their mechanical behavior from very hard and stiff (elastic moduli 4 GPa), through semi-hard and ductile, to very soft and elastic (elastic moduli 0.006 GPa). The mechanical properties were characterized in macroscale (dynamic mechanical analysis; DMA), microscale (quasi-static microindentation hardness testing; MHI) and nanoscale (quasi-static and dynamic nanoindentation hardness testing; NHI). The *stiffness-related properties* (i.e., storage moduli, indentation moduli and indentation hardness at all length scales) showed strong and statistically significant mutual correlations (all Pearson′s correlation coefficients *r* > 0.9 and corresponding *p*-values < 0.001). Moreover, the relations among the stiffness-related properties were approximately linear, in agreement with the theoretical prediction. The *viscosity-related properties* (i.e., loss moduli, damping factors, indentation creep and elastic work of indentation at all length scales) reflected the stiff-ductile-elastic transitions. The fact that the macro-, micro- and nanomechanical properties exhibited the same trends and similar values indicated that not only dynamic, but also quasi-static indentation can be employed as an alternative to well-established DMA characterization of polymer networks.

## 1. Introduction

Indentation methods have become a common characterization technique for all types of materials, including metals and alloys [1], inorganic materials and ceramics [2], and later also to viscoelastic synthetic polymers, soft polymer hydrogels, and biological tissues [3,4,5,6]. The indentation methods have a common principle: A hard indenter with known mechanical properties is forced into a flat surface of a specimen. The properties of the specimen are deduced from the size of the imprint or from the indenter penetration depth measured as a function of loading force and time [7,8].

From a historical perspective, indentation was connected with the determination of *hardness* (*H*). The first, semi-quantitative studies of the hardness date back to the 17th century. A well-known 10-step scratch hardness scale for minerals was defined by F. Mohs in 1812. The majority of classical, non-instrumented indentation devices for measurement of various types of hardness (according to Martens, Shore, Rockwell, Brinnell, etc.) were developed in the first decades of the previous century [9]. In 1951, Tabor [1] showed that the hardness of plastic materials is approximately three times higher than their macroscopic yield stress (*H* ≈ 3*Y*). Since the 1960s, Balta-Calleja and others started to investigate microhardness on polymer materials [10]. Since the 1970s, instrumented indentation devices, which record loading force as a function of penetration depth and time, are being developed. The instrumented measurements have extended the possibilities of classical indentation methods in the following three areas: (i) measurements at very low loading forces (nanoindentation), where the imprints of the indenter are too small to be detected by non-instrumented devices [11], (ii) determination of further mechanical properties, such as indentation modulus and indentation creep [2,12] and (iii) studies of viscoelastic materials, such as synthetic polymers and biopolymers, for which the classical measurements of imprints is difficult, imprecise or even impossible due to elastic recovery [4,13].

In spite of all progress described in the previous paragraph, some problems in the field of micro- and nanoindentation have not been completely elucidated. The recent review [11] concluded that just a few studies compared the hardness of materials at all length scales, i.e., at macro-, micro- and nanoscale. Moreover, most of the indentation studies have been focused on inorganic materials, such as metals and ceramics, while synthetic polymers and biopolymers have been investigated less often [3,4,14]. Finally, the micro- and/or nanoindentation studies of polymers were usually focused on some specific type of polymer materials, such as semicrystalline polymers [15], polymer nanocomposites [16] or polymer hydrogels [5] whose mechanical properties varied in a limited range. Consequently, we did not find any systematic study focused on a detailed comparison of macro-, micro- and nanomechanical properties of synthetic polymers with variable properties, changing from very hard to very soft.

**Figure 1:**
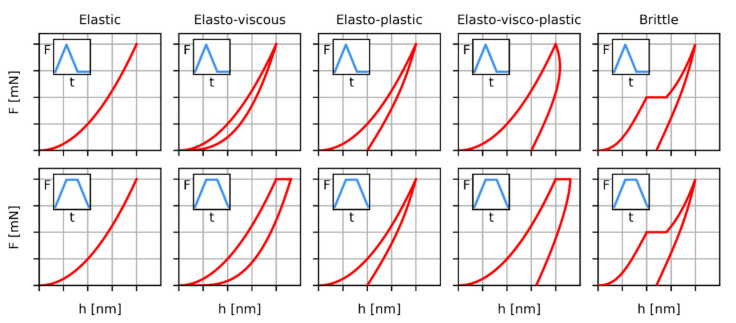
Schematic shapes of load–displacement curves (*F*-*h* curves) for five groups of materials according to their dominant mode of deformation during indentation experiments. The upper and lower rows show the *F*-*h* curves during triangular and trapezoidal loading, respectively. For the sake of clarity, the *F*-*t* curves that represent the loading are shown in small inset of each plot.

There are several reasons why indentation studies on polymers are more challenging in comparison with metals, ceramics and other inorganic materials. In general, materials can be divided into five groups according to the dominant mode of deformation during indentation experiments: elastic, brittle, elasto-plastic, visco-elastic, and elasto-visco-plastic (Figure 1). Polymer materials can exhibit all above-listed types of behavior, depending on the experimental conditions, which makes data interpretation less straightforward [13]. An additional complexity consists of the mechanical properties of polymers, which are strongly time-dependent. During the indentation experiments, hardness, modulus and other properties change not only with the hold time (or dwell time, i.e., the time of maximum load), but also with loading and unloading rate [17,18]. Moreover, the indentation properties tend to change with the maximum loading force, which is usually denoted as the indentation size effect [19,20,21,22]. In dynamic experiments the indentation moduli and damping factors depend on the frequency of force oscillations; this is analogous to dynamic mechanical analysis in macroscale [8,18,23].

This work is focused on the systematic comparison of macro-, micro- and nanomechanical properties of chemically similar crosslinked polymers with broad range of properties at room temperature. A well-known epoxy system was chosen for the investigation, the specific structure of which makes its glass transition temperature highly sensitive to the ratio of the amino-functional macro-comonomers. The polymers were based on diglycidyl ether of bisphenol A (DGEBA, the epoxy component) cured with diamine terminated polypropylene oxide (IUPAC name of the polymer: net-poly[(2,2-Bis(4-glycidyloxyphenyl)propane)-co-(polypropyleneoxide-α,ω-diamine)]; semi-trivial name: net-poly[(bisphenol A diglycidyl ether)-co-(polypropyleneoxide-α,ω-diamine)]). A mixture of two polypropyleneoxide-α,ω-diamines with different chain lengths was used as the curing component, which enabled us to tune the glass transition temperature (*T*_g_) of the complex network structure (as depicted in the Supplementary Materials) between +60 and −23 °C. Thus, at laboratory temperature (25 °C), the consistence of the samples changed from very hard and stiff (glassy), through softer and ductile ("leather-like”), to soft and elastic (rubber). Even the glassy sample, however, was a high-temperature elastomer (rubber) above its *T*_g_. The vitreous consistency of the glassy samples was caused by Van der Waals forces (similar to frozen linear polymers) and not by a fully rigid covalent network. The tested polymer networks have a bimodal structure due to the used mixture of amino components, but eventual fluctuations of crosslinking density were found to be of very small scale (around 1 nm) in early literature works [24,25,26]. As the penetration depths and indentation areas′ size were in the range of micrometers, the samples could be regarded as ideally homogeneous for the purpose of the experiments in this work. Additional information about network homogeneity can be found in the Supplementary Materials.

The samples were characterized by dynamic mechanical analysis (DMA; characterization in macroscale), by quasi-static microindentation experiments (in microscale), and by both quasi-static and dynamic nanoindentation experiments (in nanoscale). In quasi-static measurements, we evaluated not only the indentation hardness (*H*_IT_; regarded as a primary output from indentation measurements), but also indentation modulus (*E*_IT_; the secondary output), and further, supplementary properties: indentation creep (*C*_IT_) and elastic part of the indentation work (*η*_IT_). We asked the following questions: Are all the techniques applicable to the characterization of a series of polymer materials with so different properties? Are there correlations between the results at all length scales? Are the quasi-static techniques able to characterize very soft rubbery samples, for which dynamic methods are preferred? Are the supplementary quasi-static indentation properties (i.e., *C*_IT_ and *η*_IT_) relevant for the characterization of polymer materials?

## 2. Theoretical Background

### 2.1. Static, Quasi-Static and Dynamic Indentation Experiments

**Figure 2:**
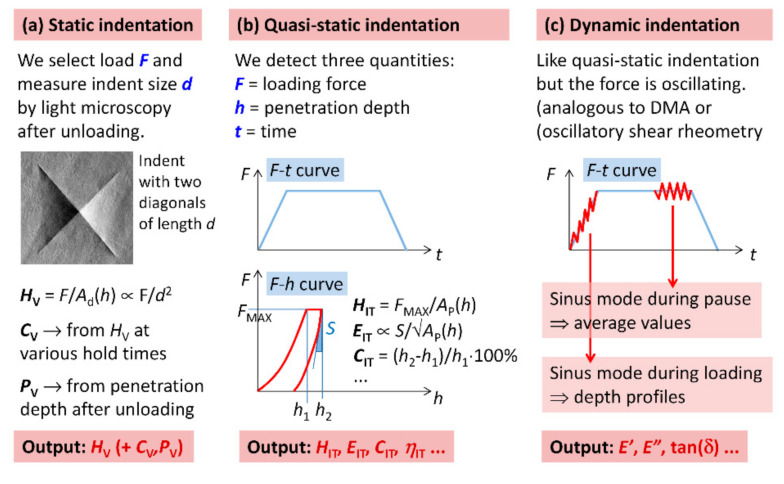
Scheme showing three basic types of indentation experiments (static, quasi-static, and dynamic) and the most important micro- or nanomechanical properties that can be obtained from the experiments. Typical static indentation experiments yield microhardness (*H*_v_) and special types of experiments can also provide microcreep (*C*_v_) and microplasticity (*P*_v_). Quasi-static indentation experiment yield indentation hardness (*H*_IT_), indentation modulus (*E*_IT_), and several supplementary properties, such as indentation creep (*C*_IT_) and elastic part of the indentation work (*η*_IT_). Dynamic indentation experiments yield parameters analogous to macroscopic dynamic mechanical analysis: storage modulus (*E*′), loss modulus (*E*″), and damping factor (tan(δ)).

There are three basic types of indentation experiments: *static*, *quasi-static*, and *dynamic* (Figure 2), and two basic types of indentation testers: *non-instrumented* and *instrumented* [9]. The non-instrumented devices determine the value of hardness from the size of the imprint on the specimen surface after unloading (static measurements; Figure 2a). The instrumented devices record penetration depth as a function of loading force (quasi-static measurements; Figure 2b), which can even oscillate with defined frequency (dynamic measurements, analogous to oscillatory shear rheometry; Figure 2c). In both quasi-static and dynamic measurements, the mechanical properties are deduced from the recorded load–displacement curves.

The characteristic output from the *static indentation experiments* (Figure 2a) is the value of hardness. There are several kinds of hardness, depending on the indenter shape and/or indentation device [8,9]. For polymers, the great majority of results come from the Vickers method (indenter is a square diamond pyramid with the angle between two non-adjacent faces = 136 degrees; [10]). Therefore, typical studies using static indentation on polymers report Vickers microhardness (*H*_v_). Special types of static experiments can yield also the creep constant (microcreep, *C*_v_) and information about elastic recovery (microplasticity, *P*_v_) [15,27,28]. The static indentation experiments are usually not performed in the nanoscale (due to the small size of imprints, which makes the measurements impractical and/or imprecise) and cannot be used for highly elastic polymer materials (due to elastic recovery, which makes the imprints hardly visible).

The characteristic output from the *quasi-static indentation experiments* (Figure 2b) is the values of the indentation hardness (*H*_IT_) and indentation modulus (*E*_IT_), which are usually determined according to Oliver and Pharr theory (O&P; [2]) that is included also in the ISO 14577 standard. It is worth noting that the very successful O&P theory was derived for elasto-plastic materials [2,29], while polymers are elasto-visco-plastic. Nevertheless, the evaluation of micromechanical properties of polymers from quasi-static experiments in terms of O&P theory is feasible (as evidenced by many previous studies [30,31,32,33]), but it makes the final values of micromechanical properties more sensitive to the experimental conditions and viscosity-related effects. For example, due to the creep and pile-up effects, the *H*_IT_ values of polymers tend to be somewhat higher than *H*_V_ from non-instrumented experiments [34,35] and the *E*_IT_ values tend to be higher than macroscale Young′s modulus [3,36]. The above-mentioned standard (ISO 14577) also defines other, supplementary quantities that can be obtained from quasi-static indentation, namely indentation creep (*C*_IT_) and the ratio of elastic to total work of indentation (*η*_IT_). Finally, the standard defines Martens hardness (*H*_M_; also known as universal hardness), whose calculation is *independent* on O&P theory, and reduced modulus (*E*_r_), whose calculation does not include Poisson′s ratio of the polymer.

Characteristic output from the *dynamic indentation experiments* (Figure 2c) is the storage and loss moduli (*E*′ and *E*″) and damping factor (tan(δ)). These properties (*E*′, *E*″, and tan(δ) = *E*″/*E*′) are analogous to the properties from oscillatory shear rheometry, i.e., to the storage and loss moduli measured in shear, together with damping factor measured in shear (*G*′, *G*″, and tan(δ) = *G*″/*G*′). Therefore, the dynamic properties characterize viscoelasticity of the investigated material: the storage moduli (*E*′ or *G*′) represent stiffness and elasticity, the loss moduli (*E*″ or *G*″) represent flow and viscosity, and the changes of damping factors (tan(δ)) indicate viscoelastic transitions, such as a glass transition temperature or gel formation [23]. The indentation moduli (*E′* and *E″*) can be recalculated to shear moduli (*G*′ and *G*″) using a simple formula from polymer physics (*E* = 2*G*(1 + *ν*), where ν is Poisson′s ratio [32,37,38]). It is also possible to calculate the absolute value of complex shear modulus (|G*| = √(*G*′^2^ + G″^2^)), which represents overall material resistance to the shear deformation [23]. The oscillations of loading force during dynamic indentation experiments can be applied in two ways, as schematically shown in Figure 2c: (i) during the increasing loading force (this is occasionally called *sinus mode during loading*) or (ii) during the time when loading force is kept constant (this is occasionally called *sinus mode during pause*). *Sinus mode during loading* gives viscoelastic properties as a function of penetration depth, while *sinus mode during pause* is usually applied after some time of maximum load (in order to minimize creep-related effects) and gives viscoelastic properties at given load and penetration depth. In this contribution, all dynamic indentation experiments were performed in the *sinus mode during pause*.

### 2.2. Stiffness-Related Properties of Amorphous and Crosslinked Polymers

#### 2.2.1. Basic Formulas for Stiffness-Related Properties of Polymers

It has been shown that the stiffness-related properties of polymers, such as elastic modulus (*E*), shear modulus (*G*), yield stress (*Y*) and hardness (*H*) are closely connected [39,40]. Tabor [1] found that the basic micromechanical property, *H*, is proportional to the macroscopic yield stress:

(1) H≈3Y

The approximate symbol in Equation (1) indicates that the relation was derived for ideally plastic materials (such as some metals and alloys), while for elasto-visco-plastic materials (such as polymers and their composites) it is just the first approximation. Struik [41] derived another approximate formula for amorphous polymers:

(2) E≈30Y

which indicates that the polymer systems with high elastic modulus usually exhibit also high yield stress and *vice versa*. Although the value of proportionality constant in Equation (2) (i.e., the value of 30) can vary in a quite broad range, the approximate linear relation between elastic modulus and yield stress (or hardness, because *H* ≈ 3*Y* according to Equation (1)) was verified experimentally not only for amorphous polymers, but also for semicrystalline polymers [41,42], rubbers [43,44], and crosslinked polymers [45,46]. Furthermore, textbooks [37,38] give a formula connecting tensile modulus (*E*) and shear modulus (*G*):

(3) $$E\approx 2G\left(1+\nu \right)$$

where *ν* is Poisson′s ratio (typical values of Poisson′s ratio for polymer materials range from 0.3 to 0.5). If the shear modulus is determined from oscillatory rheometry, then Equation (3) holds for storage modulus (*G*′) that reflects elastic properties, whereas loss modulus (*G*″) is connected with viscous properties [23]. Combination of Equations (1)–(3) gives the final simplified but very useful formula illustrating mutual proportionality among the stiffness-related properties of polymer systems:

(4) $$H\approx 3Y\approx E/10\approx \left[2G\left(1+\nu \right)\right]/10$$

It is worth noting that exact numerical values of all constants in Equation (4) may differ for different studies, as the constants are inevitably dependent on experimental conditions, such as: (i) the type of measurement of elastic modulus, *E* (tensile moduli tend to be lower than compressive moduli; the same applies to yield stresses, *Y*), (ii) the frequency at which shear modulus *G* is determined from dynamic measurements (higher frequencies result in higher storage moduli), and (iii) the type of indentation measurement, which influences *H* (various types of hardness exhibit different absolute values, the hold time at maximum load decreases hardness due to creep effects, and the selected loading force may influence the result due to possible indentation size effects). 

Despite the fact that the values of constants in Equation (4) are approximate, the linear relations among all stiffness-related properties (*H*, *Y*, *E*, and *G*) usually hold very well *for given series of polymer samples and given set of experimental conditions*. For example, the linear *H*-*Y*-*E*-*G* relations were observed for series of linear amorphous polymers with increasing *T*_g_ or a semicrystalline polymer with increasing crystallinity; references are summarized in a classical textbook about the microhardness of polymers [39,40]. Furthermore, numerous studies have confirmed that the linear *H*-*Y*-*E*-*G* correlations hold also for series of crosslinked polymers (i.e., for moderately crosslinked *rubbers*, highly crosslinked *thermosetting resins* and physically crosslinked *thermoplastic elastomers*) on condition that the authors did not change too many parameters within the studied system; selected publications dealing with stiffness-related properties of crosslinked polymers have already been cited above [43,44,45,46]. Moreover, the linear proportionality between the stiffness-related properties was observed also for multiphase, multicomponent polymer systems, such as polymer blends with an increasing amount of one component [32,42,47] and polymer composites with an increasing amount of filler [18,44,48,49]. Some limitations of our approximations are discussed below (Section 5.1) and in the Supplementary Materials, but we can conclude that Equation (4) shows the key trend for the stiffness-related properties of polymer systems, which is the strong correlation among hardness, yield stress, and elastic moduli.

#### 2.2.2. Stiffness-Related Properties of Crosslinked Polymers

Crosslinked polymers represent an ideal model system to study relations among stiffness-related properties in macro-, micro- and nanoscale, which should be approximately proportional according to Equation (4). The stiffness of polymer networks can be controlled in a very broad range by changing crosslinking density. In our specific case of carefully synthesized epoxy resin networks, the materials are highly homogeneous and the crosslinking density can be fine-tuned by small changes of the ratio of the components (as evidenced in Section 3.1 and Section 3.2 below). Consequently, we get the series of materials with very similar molecular structures (all components are the same, only their ratio and crosslinking density changes) and identical morphology (all systems are homogeneous within all studied length scales). This minimizes all unwanted effects and mechanical properties are expected to change monotonously due to the crosslinking density.

**Figure 3:**
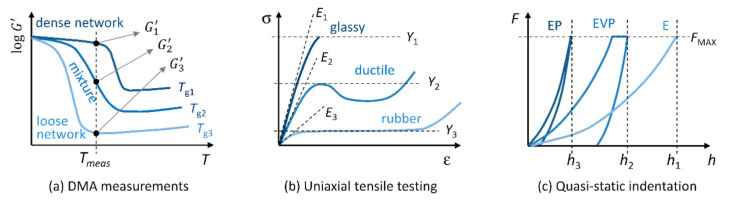
The scheme showing how the change of crosslinking density influences properties of crosslinked polymers measured by (**a**) DMA, (**b**) uniaxial tensile testing and (**c**) quasi-static indentation. The polymer with the highest crosslinking density (and the highest *T*_g_) is marked with the darkest blue color, the polymer with the lowest crosslinking density (and the lowest *T*_g_) is marked with the lightest blue color, and the mixture of the two polymers (with intermediate *T*_g_) is marked with medium blue color.

Figure 3 is a compilation from textbooks on polymer physics and indentation [8,50,51] that illustrates how the change of crosslinking density influences mechanical properties measured by DMA (Figure 3a), by uniaxial tensile testing (Figure 3b), and by indentation testing (Figure 3c). DMA curves (Figure 3a) demonstrate the correlations among crosslinking density, glass transition temperature and stiffness: A polymer with low crosslinking density (light blue line) has *T*_g_ below the temperature of measurement and exhibits very low modulus, while a polymer with high crosslinking density (dark blue line) has *T*_g_ above the temperature of measurement and exhibits very high modulus. Tensile curves (Figure 3b) of the same polymers show that the polymer with the highest crosslinking density is glassy and brittle, the intermediate polymer is ductile and the polymer with the lowest crosslinking density behaves like elastic rubber. Indentation curves (Figure 3c) illustrate that the highly-crosslinked polymer is expected to exhibit elasto-plastic behavior (EP), intermediate polymer is elasto-visco-plastic (EVP), and the polymer with the lowest crosslinking density is entirely elastic (E).

The increase in the storage modulus with increasing *T*_g_ is quite a general trend that holds *within given polymer system* (if we change the individual components or overall morphology, the correlation between stiffness and *T*_g_ may be lost). Due to Equation (4), the trend is expected to hold not only for the storage modulus, but for all stiffness-related properties (i.e., *H*, *Y*, *E*, and *G*). However, the increase in the stiffness-related properties with *T*_g_ is not linear in the whole range of glass transition temperatures. This is clearly visible from the shape of DMA curves in Figure 3a: The small change of *T*_g_ or temperature of measurement may result in large shifts of storage modulus. The combination of Equation (4) with the fact that stiffness-related properties of amorphous polymers depend on *T*_g_ can be written as follows:

(5) $$H\approx 3Y\approx E/10\approx \left[2G\left(1+\nu \right)\right]/10\approx f\left(T_{g}\right)$$

where *f*(*T*_g_) is an increasing function of *T_g_*. The exact shape of function *f*(*T*_g_) depends on the investigated system, but for some specific types of amorphous and/or crosslinked polymers, the shape of *f*(*T*_g_) is known. These special cases are discussed in more detail in Supplementary Materials for the sake of completeness. The key conclusion for this study is that the stiffness-related properties of crosslinked polymers are predicted to increase monotonously with crosslinking density and *T_g_*.

## 3. Experimental

### 3.1. Materials

The poly(oxypropylene) diamines *Jeffamine D2000* (*M*_n_ = 1968 g/mol) and *Jeffamine D400* (*M*_n_ = 432 g/mol), as well as the epoxide Diglycidyl ether of Bisphenol A (*DGEBA*, 99.7% pure, *M*_n_ = 340.9 g/mol), were purchased from Sigma-Aldrich (St. Louis, MO, USA) and used as obtained.

### 3.2. Preparation of Polymer Networks with Increasing Glass Transition Temperature

The samples of epoxy resin networks (common literature name: diglycidyl ether of bisphenol A cured with diamine terminated polypropylene oxide; IUPAC name: net-poly[(2,2-Bis(4-glycidyloxyphenyl)propane)-co-(polypropyleneoxide-α,ω-diamine)]; semi-trivial name: net-poly[(bisphenol A diglycidyl ether)-co-(polypropyleneoxide-α,ω-diamine)]) were synthesized according the procedure published elsewhere [52]. In these networks, the elastic chains were part of the amino-component(s) and the ratio of the long (D2000) to the short (D400) elastic (amino-functional) chains was varied in order to fine-tune the glass transition temperature (*T*_g_) (Table 1): from pure Jeffamine D400-cured networks (short chains only) to pure Jeffamine D2000-cured networks (long chains only). The second, epoxy component of the networks (DGEBA) was molten and subsequently cooled back to room temperature (it stays in the form of undercooled liquid at RT up to several weeks). Thereafter the appropriate amount(s) of the Jeffamine D component(s) (D2000 and/or D400) was/were added (their ratios are summarized in Table 1) and the materials were thoroughly mixed, put into a mold (made of *Teflon*^®^; Chemours, Wilmignton, DE, USA) and cured at 120 °C for 3 days.

The samples after curing were highly homogeneous as indicated by three facts: At first, the visual check of the samples confirmed that they were all transparent, clear, and without any visible inhomogeneities or bubbles. At second, the samples exhibited just one *T*_g_, which confirmed that the two components were miscible, not forming separate domains [53]. Third, the samples were measured by three independent methods (DMA, microindentation, and nanoindentation) and the strong correlation between stiffness-related properties at all length scales was achieved (as evidenced below), although each method used a different piece of the prepared specimen. More details about the structure and homogeneity of the prepared samples can be found in Supplementary Material.

The samples after curing were used for all subsequent experiments: DMA measurements employed directly the specimens from the molds, micro- and nanoindentation measurements were made on the smooth surfaces coming from the bottom of the molds.

### 3.3. Dynamic Mechanical Analysis

Dynamic-mechanical thermal analysis (DMA) of all resins was carried out on rectangular platelet-shaped samples, using an advanced multi-functional rheometer ARES-G2 (from TA Instruments, New Castle, DE, USA—part of Waters, Milford, MA, USA). An oscillatory shear deformation at the constant frequency of 5 Hz and at the heating rate of 3 °C/min was applied, and the temperature dependences of the storage shear modulus and loss modulus as well as of the damping factor (G′, G″ and tan(δ), respectively) were recorded. The initial value of the oscillatory shear deformation was set at 0.01% and an “Auto-Strain” function was applied (see example in Supplementary Material), which upon softening of the material (if torque value fell below 2000 μN·m) caused a gradual increase in the deformation amplitude, maximally up to the limit value of 4%, which was never exceeded (it was verified, that the linear deformation region in the studied samples extends at least up to 20%). The Auto-Strain function was used in order to obtain good quality data points in the rubbery region, where higher deformations are required. The temperature range of the analyses was typically from −90 to +100 °C. The geometry of the deformed part of all the tested specimens was always the same: 20 mm height (height of the whole specimen: 40 mm), 10 mm width, and 1 mm thickness.

### 3.4. Microindentation

Instrumented microindentation hardness testing (MHI) of all samples was measured with micro-combi tester MCT (CSM Instruments, Corcelles, Switzerland). All experiments were performed in the quasi-static mode: linear loading rate 3000 mN/min, hold time 30 s at maximum loading force 400 mN, and linear unloading rate 3000 mN/min. The indentations were made into the smooth bottom surface from the mold. The size of the specimen for microindentation was approximately 10 mm × 10 mm × 1 mm. The size of the indents varied from ca 70 μm (for the hardest samples) to ca 800 μm (for the softest samples). For each specimen, at least 15 indentations were measured and the results were averaged. The evaluated micromechanical properties were: indentation hardness (*H*_IT_), indentation modulus (*E*_IT_), indentation creep (*C*_IT_), elastic part of the indentation work (*η*_IT_), and Martens hardness (*H*_M_, also referred to as universal hardness). The calculations of *E*_IT_ and *H*_IT_ were based on Oliver and Pharr’s theory [2], while *H*_M_ was calculated directly from experimental data [7]. The basic principle of *H*_IT_, *E*_IT_ and *C*_IT_ calculation is shown in Figure 2 and more details can be found elsewhere [7,12]. All calculations were performed according to ISO 14577 standard. Poisson ratio *v* = 0.3 was used for all calculations in this work.

### 3.5. Nanoindentation

All nanoindentation experiments were performed using the Nanoindentation Tester (NHT^3^, Anton Paar, Corcelles, Switzerland) with a modified Berkovich indenter. The measurements were done in force control mode to a maximum force F_max_ of 100 mN with a loading rate of 750 mN/min (loading time 8 s). The indentations were made into the smooth bottom surface from the mold (like in the case of microindentation). The size of the specimen for nanoindentation was approximately 10 mm × 10 mm × 1 mm. The size of the indents varied from ca 30 μm (for the hardest samples) to ca 400 μm (for the softest samples). Two types of measurement protocol were used: (1) quasistatic, with the hold period of 30 s at the F_max_, followed by unloading at the rate of 750 mN/min and (2) dynamic, with a hold period of 60 s at F_max_ to accommodate for creep. The 60 s long hold period was followed by dynamic oscillations at a frequency of 5 Hz and full amplitude of 10 mN for 30 s. After the dynamic oscillations, the indenter was fully unloaded at a rate of 750 mN/min. The parameters of the quasistatic and dynamic NHI were as close as possible to the parameters of quasistatic MHI and the macroscale DMA tests. The quasistatic indentations were analyzed using ISO 14577 standard to obtain hardness (*H*_IT_), elastic modulus (*E*_IT_), indentation creep (*C*_IT_) and the ratio of elastic to total work of indentation (*η*_IT_). The dynamic indentations were analyzed using a Kelvin–Voigt model to obtain storage and loss moduli as well as tangent delta [38]. The main output of the dynamic measurements was storage modulus (*E*′), loss modulus (*E*″) and damping factor (tanδ; also known as loss factor). For the sake of feasible comparison of NHI data with corresponding DMA data, we recalculated the storage and loss indentation moduli to corresponding shear moduli (*G*′ = *E*′/[2(1 + *ν*)], *G*″ = *E*″/[2(1 + *ν*)], where *ν* is a Poisson ratio). Damping factors tanδ remained the same (because tanδ = E″/E′ = G″/G′). Poisson ratio *v* = 0.3 was used for all calculations in this work. 

### 3.6. Statistical Evaluation of Results

Mechanical properties determined for all individual samples from all methods (DMA, MH and MHI) were collected in a spreadsheet program (MS Excel) and basic statistical evaluation (calculation of means and standard deviations) was performed. Final statistical plots, correlation coefficients and *p*-values were calculated by means of user-defined scripts in Python programing language, using its freeware modules for data manipulation, plotting and statistical processing [54]. The Python scripts, which were used for the calculations, are available on request to the corresponding author of this work. The statistical graphs (scatterplot matrix graphs and correlation matrix tables in the form of heatmaps) are described directly in the Results section. The *Pearson correlation coefficients* (frequently denoted as Pearson′s *r* or just *r*) were used to quantify the strengths of expected linear correlations between selected mechanical properties. Briefly, Pearson′s *r* ranges from +1 to −1, where +1 means total positive linear correlation, −1 means total negative linear correlation, and 0 means no linear correlation. The *coefficients of determination* (*R*^2^) were used as the overall measure of fit quality; in the case of simple linear regressions used in this study, *R*^2^ ranges from 0 (bad fit) to +1 (perfect fit) and equals to square of Pearson′s correlation coefficient (*R*^2^ = *r*^2^). The *probability values* (also referred to as *p*-values or just *p*) gave the probability that for our statistical model (here: correlation between mechanical properties) we would have obtained the same or stronger result just by coincidence (here: that we would have obtained the same or stronger correlation if the data were random). The results were considered statistically significant if the *p*-value was below 0.05 (i.e., below significance level α = 5%; this is a common limit in statistical analyses). More details about the statistical graphs and coefficients can be found elsewhere [55].

## 4. Results

### 4.1. Quasi-Static Experiments

**Figure 4:**
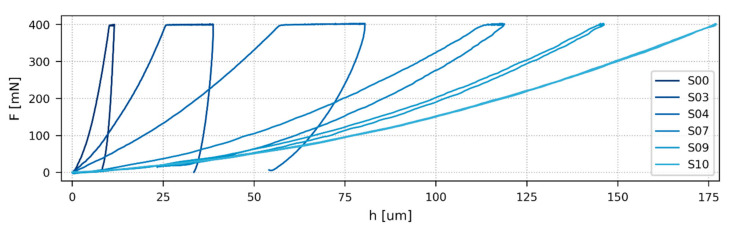
*F*-*h* curves of selected samples from quasi-static microindentation hardness testing. The darkest colors correspond to the highest *T*_g_′s (see Table 1 for sample description and *T*_g_′s).

**Figure 5:**
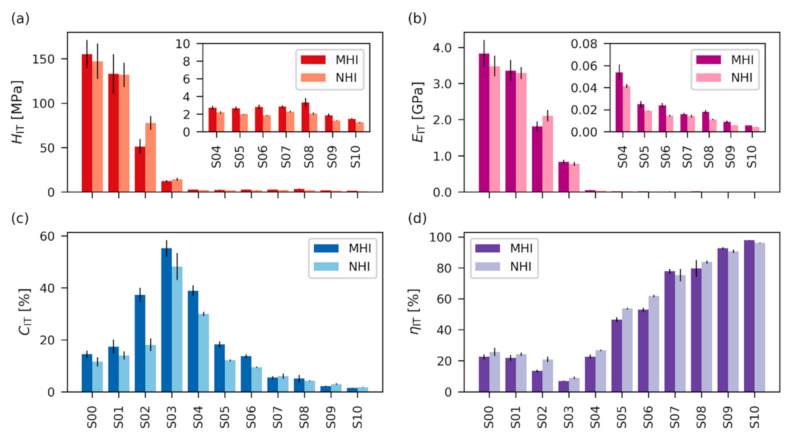
Results of quasistatic experiments: quasistatic instrumented microindentation (MHI) and quasistatic instrumented nanoindentation (NHI): (**a**) indentation hardness, *H*_IT_, (**b**) indentation modulus (*E*_IT_), (**c**) indentation creep (*C*_IT_), and (**d**) elastic part of the indentation work, *η*_IT_.

Quasi-static experiments were performed in microscale (quasi-static microindentation, Section 3.4) and nanoscale (quasi-static nanoindentation; Section 3.5). Figure 4 shows typical *F*-*h* curves from microindentation experiments. They illustrate how the dominant mode of deformation changed with *T*_g_ (compare Figure 1 and Figure 3). The polymer networks with the highest *T*_g_ (such as S00) were mostly elasto-plastic, the networks with *T*_g_ close to the temperature of measurement (such as S03–S07) were elasto-visco-plastic, the networks with *T*_g_ below the temperature of measurement (such as S09) were elasto-viscous, and the networks with very low *T*_g_ (S10) were completely elastic rubbers. The micro- and nanomechanical properties changed accordingly, as summarized in Figure 5. The indentation hardness (Figure 5a) decreased steeply with *T*_g_ for the first four, macroscopically stiff vitrified networks (samples S00–S03) and then it decreased slowly for the remaining seven, macroscopically soft elastic networks (samples S04–S10). The indentation modulus (Figure 5b) showed a very similar trend like the indentation hardness, which was in good agreement with Equation (4) which predicts approximately linear proportionality *E* ≈ 10*H*. The indentation creep (Figure 5c) was relatively low for the stiffest, elasto-plastic samples with the highest *T*_g_ (S00–S01), exhibited a maximum for intermediate, elasto-visco-plastic samples (peak for sample S03), and then decreased for samples with mostly elasto-viscous behavior (S07–S09), down to almost zero for the completely elastic rubber (S10). The elastic part of the indentation work (Figure 5d) showed an inverse behavior to *C*_IT_: The stiffest samples showed certain elasticity (behavior of “*enthalpic springs*”, see Section 5.1 below), the softest samples exhibited maximal elasticity (behavior of “*entropic springs*”, see Section 5.1 below), and the intermediate samples showed minimal elasticity (minimum for sample S03).

It is worth noting how the changes of *F*-*h* curves in Figure 3 corresponded to the final micro- and nanomechanical properties in Figure 4: *H*_IT_ decreased with the maximum penetration depth (*h*). *E*_IT_ was proportional to the slope at the beginning of the unloading curve (*S* in Figure 2b). *C*_IT_ increased with the difference between the penetration depths at the beginning and at the end of maximal loading (*h*_1_ and *h*_2_ in Figure 2b). Finally, *η*_IT_ was linked to the difference between loading and unloading curves—the smaller the difference, the higher the elasticity of the specimen.

The results from the quasi-static microindentation experiments (MHI) and quasi-static nanoindentation experiments (NHI) were in very good agreement for all four quantities (*H*_IT_, *E*_IT_, *C*_IT_ and *η*_IT_) as evidenced in Figure 5. The overall trends were almost identical and the small discrepancies between individual values could be attributed to experimental errors. Tables with numerical values and standard deviations of all quasi-static micro- and nanomechanical properties are given in Supplementary Materials.

### 4.2. Dynamic Experiments

**Figure 6:**
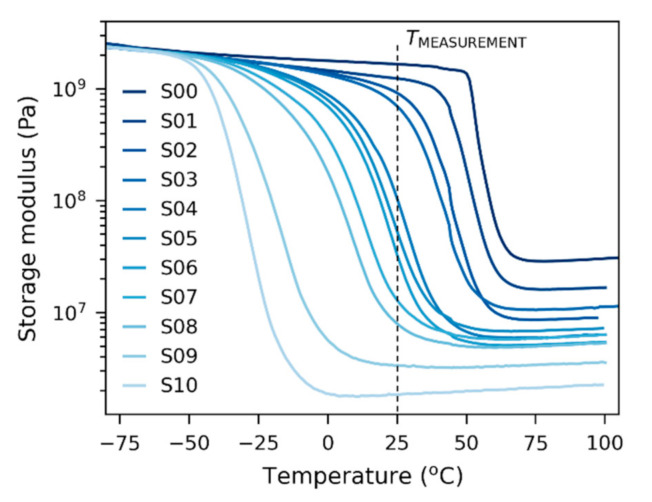
*G*′-*T* curves from macroscale dynamic mechanical analysis (DMA) testing of all studied samples, illustrating the increase in storage modulus with the increasing glass transition temperature.

The dynamic experiments were performed in macroscale (DMA, Section 3.3) and nanoscale (dynamic NHI; Section 3.5). Figure 6 shows the direct output from macroscale DMA measurements—the storage moduli of all samples as a function of temperature. The figure illustrates the range of properties achieved by the variation of the ratio of the macro-comonomeric amino components (D400 and D2000) in the polymeric networks. Due to their somewhat complex structure shown in the Supplementary Materials, the increase in the content of the short-chain amino component (D 400) leads to the increase in both glass transition temperature and crosslinking density (higher moduli in the “rubbery plateau” at high temperatures, after the glass transition step). The increase in the crosslinking density was associated with the fact that the number of shorter chains per volume unit was higher in comparison with longer chains (which resulted in the formation of more crosslinks with DGEBA units). The increase in the glass transition temperature (*T*_g_) with a higher concentration of short chains was associated with higher the fraction of the semi-rigid sub-structures in the network (based on chains of DGEBA connected by N-atoms from the amines, see scheme in the Supplementary Materials); this led to the reduced segmental mobility of the whole network. This property of DGEBA-diamine networks was deliberately used in this work to synthesize a quasi-continuous series of chemically very similar networks with continuously varied *T*_g_ (while the modulus in the rubbery region also changed, which did not pose a problem). Crosslinking density could be evaluated from the moduli in the rubbery range and compared with theoretical values calculated from the volume concentration of the diamine chains (see the additional information in the Supplementary Materials).

**Figure 7:**
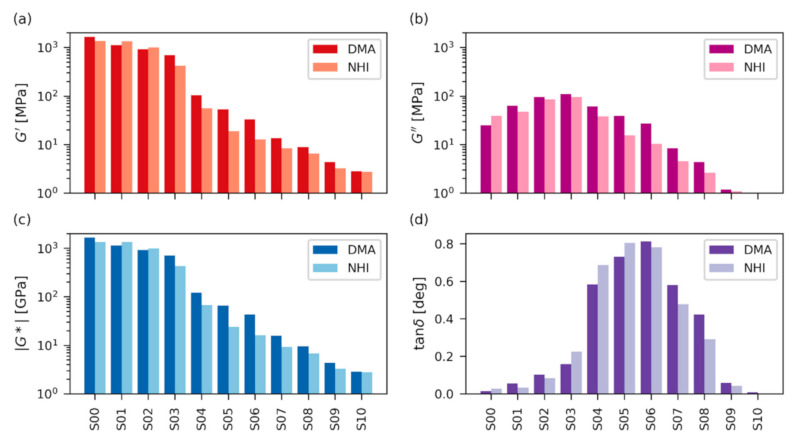
Results of dynamic experiments: macroscale dynamic mechanical analysis (DMA) and dynamic nanoindentation experiments (NHI): (**a**) storage modulus, *G*′, (**b**) loss modulus, *G*″, (**c**) absolute value of complex modulus |G*|, and (**d**) damping factor, tanδ.

The final processed data from both macro- and nanoindentation dynamic experiments are summarized in Figure 7; all moduli from dynamic experiments are plotted in logarithmic scale, which is typical for DMA results [23]. The storage modulus from dynamic experiments (*G′*; Figure 7a) showed similar trends to the indentation modulus and hardness in quasi-static experiments (Figure 5a,b): The storage moduli of the first four, macroscopically stiff samples with higher *T*_g_ (S00–S03) were high (*G*′~10^3^ MPa), while the storage moduli of the remaining seven, macroscopically soft samples with lower *T*_g_ (S04–S10) were at least one order of magnitude lower (*G*′ ≤ 10^2^ MPa). We note that the indentation modulus and hardness (Figure 5a,b) showed the same clear difference between the first four stiff and the last seven soft samples. Loss modulus (*G*″; Figure 7b) was lower than the storage modulus for all samples, which confirmed that for every sample the solid-like, elastic behavior (represented by *G*′) predominated over liquid-like, viscous behavior (represented by *G*″). The highest values of G″ were observed for samples around S03, which corresponded very well to the quasi-static measurements: Sample S03 showed the highest viscous contribution (Figure 4) on *F*-*h* curves and, consequently, it exhibited the highest creep (Figure 5c) and the lowest elasticity (Figure 5d). Absolute value of complex modulus (|G*|; Figure 7c) represents overall resistance of material to both elastic and viscous deformation (as follows from its definition: |G*| = |*G*′ + i*G*″| = √(*G*′^2^ + *G*″^2^)). In our case |G*| showed an almost identical trend to *G*′ because the contribution of storage modulus was dominating for all samples, confirming their solid-like behavior. The value of damping factor (tanδ; Figure 7d) was low for the samples with the highest *T*_g_ (S00–S03), then it steeply increased for the samples with lower *T*_g_ (S04–S08), and finally, it decreased for samples with the lowest *T*_g_ (S09–S10). Again, this corresponded very well to the definition of damping factor (tanδ = *G*″/*G*′) and other experimental results: Samples S00–S03 were macroscopically stiff (with high values of *H*_IT_ and *E*_IT_—Figure 5a,b and dominating contribution of G′—Figure 7a,b). Samples S04–S08 were macroscopically soft and ductile (with low values of *H*_IT_ and *E*_IT_ combined with high *C*_IT_ and moderate *η*_IT_—Figure 5, and relatively high contribution of G″—Figure 7a,b). Samples S09–S10 were macroscopically soft and elastic (with low values of *H*_IT_ and *E*_IT_ combine with low *C*_IT_ and maximal *η*_IT_—Figure 5, and negligibly low contribution of G″—Figure 7a,b).

The results from the dynamic experiments in macroscale (DMA; Figure 7, darker columns) and nanoscale (dynamic NHI; Figure 7; brighter columns) were in very good agreement for all four quantities (*G*′, *G*″, |G*| and tanδ). Moreover, the macro- and nanoscale dynamic results correlated very well micro- and nanoscale quasi-static experiments, as discussed in the next section. Like in the case of quasi-static experiments, the results of dynamic experiments exhibited almost identical trends and the small discrepancies between individual values of DMA and NHI could be attributed to minor experimental errors. Tables with numerical values of all dynamic macro- and nanomechanical properties are given in Supplementary Materials.

## 5. Discussion

### 5.1. Correlations between T_g_ and Stiffness-Related Properties

**Figure 8:**
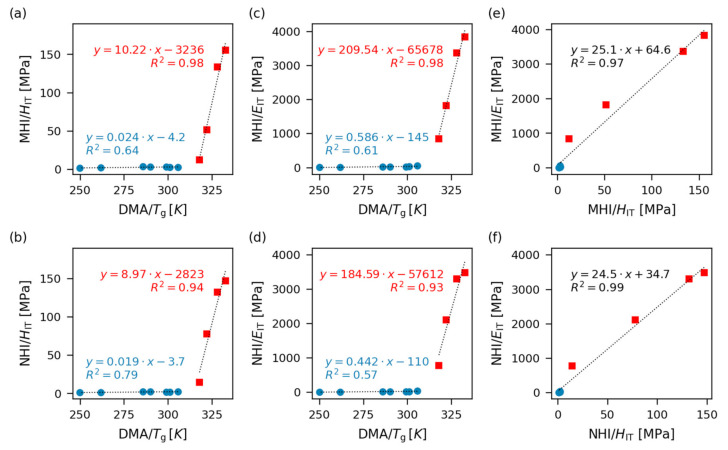
Correlations among glass transition temperature from DMA (DMA/*T*_g_), indentation hardness from micro- and nanoindentation (MHI/*H*_IT_ (**a**) and NHI/*H*_IT_ (**b**)), and indentation modulus from micro- and nanoindentation (MHI/*E*_IT_ (**c**) and NHI/*E*_IT_ (**d**)). Subfigures (**e**) and (**f**) show correlations between indentation hardness and indentation modulus from micro- and nanoindentation, respectively.

Correlations between glass transition temperature and selected micro- and nanomechanical properties are summarized in Figure 8. In agreement with theoretical predictions (Equation (5)), the stiffness-related properties (MHI/*H*_IT_, MHI/*E*_IT_, NHI/*H*_IT_, and NHI/*E*_IT_) increased with *T*_g_. However, the increase was not linear in the whole range of *T*_g_′s and we observed two distinct linear correlation regions. The first four harder samples (S00–S03; red squares in Figure 8) behaved like glassy polymers and their stiffness increased with *T*_g_ steeply, while the softer samples (samples S04–S10; blue circles in Figure 8) behaved like elastic rubbers and their stiffness increased with *T*_g_ moderately.

The reason why we observe two linear correlation regions in Figure 8a–d is the different mechanism of deformation of the polymer networks below and above their glass transition temperature. A detailed explanation is given in the Supplementary Materials, while this paragraph outlines the main idea. The crosslinked (elastomeric) polymers measured below their *T*_g_ contain polymer chains, whose movement is frozen [50]. The rigid chains cannot change their conformations and the material upon deformation behaves like an “*enthalpic spring*″, i.e., the deformation occurs just due to *small changes of intermolecular distances*, which is connected with the enthalpic contribution to the free energy of the system (Δ*G* = Δ*H* − *T*Δ*S*, where Δ*H* and Δ*S* represent enthalpic and entropic changes, respectively). The enthalpic effects originate in Van der Waals forces, which are responsible for the intermolecular attraction (as well as repulsion upon compression), and which are considerably strong between large macromolecules (this results in the high elastic moduli in the range of 10^9^ Pa). 

The crosslinked polymers measured above their *T*_g_ contain flexible (and segmentally mobile) polymer chains, whose movement is restricted mostly by crosslinks [51]. The flexible polymer chains can change their conformations and behave like an “*entropic spring*″, i.e., the chains are deformed by *large changes of dihedral angles*, which results in conformational changes connected with the entropic contribution (Δ*S*) to the free energy of the system (Δ*G* = Δ*H* − *T*Δ*S*). The entropic effects are associated with the statistical definition of entropy (*S* = *k* lnΩ; where Ω is the number of microstates): Number of conformations (i.e., microstates) corresponding to deformed and elongated polymer chains is lower than the number of conformations corresponding to undeformed polymer coils. The conformational changes below *T*_g_ require less energy in comparison with the changes of intermolecular distances above *T*_g_ and so the materials below their *T*_g_ are softer (elastic moduli in the range of 10^6^ Pa).

The theoretical justification of the observed relations between *T*_g_ and stiffness-related properties differs for crosslinked polymers below their *T*_g_ (vitrified networks; we employ the theory of the polymers in the glassy state) and above their *T*_g_ (elastic rubbers; we employ the theory of rubbery elasticity). Again, a more detailed explanation is given in Supplementary Materials, while this paragraph summarizes the main ideas. For the vitrified networks, the decisive parameter influencing their stiffness is the glass transition temperature, while the average molar mass between crosslinks (*M*_c_) is just a supplementary parameter influencing the final *T*_g_ [39,41,53]. For the elastic rubbers, the decisive parameter influencing stiffness is the crosslinking density characterized by *M*_c_ (or by the related concentration of elastically active chains *c*(EAC), while *T*_g_ is just a supplementary parameter [50,51]. The fact that the influence of crosslinking density is different for the polymer networks above and below *T*_g_ explains why the samples formed two groups in Figure 8a–d. Moreover, the strong linear correlations between indentation modulus and hardness in both micro- and nanoscale in the whole range of glass transition temperatures (Figure 8e,f, rightmost column, *R*^2^ > 0.95) were in agreement with the theoretically predicted linear correlations between stiffness-related properties within groups of similar polymers (Equations (1)–(4)).

### 5.2. Correlations among Macro-, Micro- and Nanomechanical Properties

**Figure 9:**
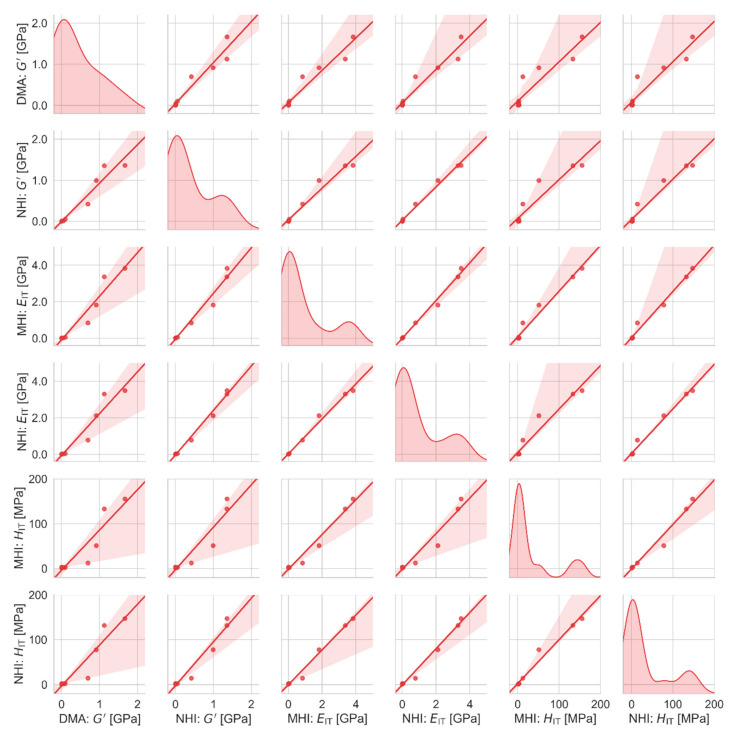
Scatterplot matrix graph showing correlations between selected stiffness-related properties in macro-, micro- and nanoscale: storage modulus from dynamic mechanical analysis (DMA/*G*′), storage modulus from nanoindentation (NHI/*G*′), indentation modulus from micro- (MHI/*E*_IT_) and nanoindentation (NHI/*E*_IT_), and indentation hardness from micro- (MHI/*H*_IT_) and nanoindentation (NHI/*H*_IT_). Diagonal elements of the scatterplot matrix graph display distributions of the individual quantities. Off-diagonal elements show linear regression between the pairs of the quantities.

**Figure 10:**
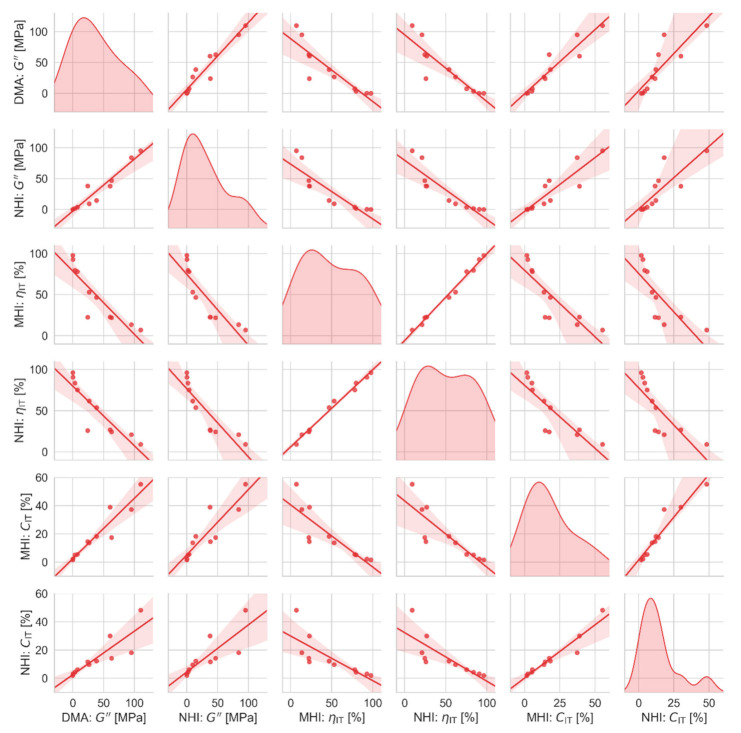
Scatterplot matrix graph showing correlations between selected viscosity-related properties in macro-, micro- and nanoscale: loss modulus from dynamic mechanical analysis (DMA/*G*″), loss modulus from nanoindentation (NHI/*G*″), elastic part of indentation work from micro-(MHI/η_IT_) and nanoindentation (NHI/η_IT_), and indentation creep from micro-(MHI/*C*_IT_) and nanoindentation (NHI/*C*_IT_). Diagonal elements of the scatterplot matrix graph display distributions of the individual quantities. Off-diagonal elements show linear regression between the pairs of the quantities.

**Figure 11:**
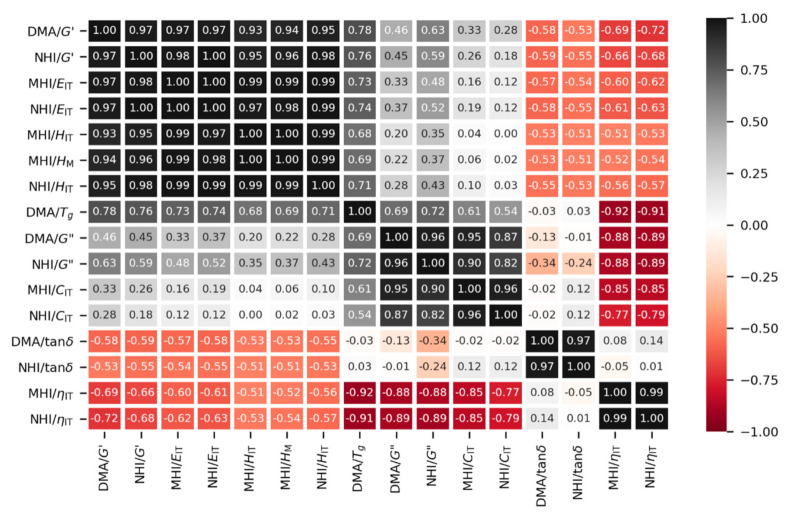
Correlation matrix table showing Pearson′s coefficients *r* for all pairs of experimentally determined properties in macro-, micro- and nanoscale. The table is presented as a heatmap (darker color means stronger correlation). The properties in the table are: storage modulus, loss modulus, damping factor and glass transition temperature from dynamic mechanical analysis (DMA/*G*′, DMA/*G*″, DMA/tan(δ) and DMA/*T*_g_), analogous properties from dynamic nanoindentation experiments (NHI/*G*′, NHI/*G*″ and NHI/tan(δ)), indentation hardness, modulus, elastic part of indentation work and creep from quasi-static microindentation experiments (MHI/*H*_IT_, MHI/*E*_IT_, MHI/η_IT_ and MHI/*C*_IT_), and analogous properties from quasi-static nanoindentation experiments (NHI/*H*_IT_, NHI/*E*_IT_, NHI/η_IT_ and NHI/*C*_IT_).

Correlations between all measured macro-, micro- and nanomechanical properties are summarized in Figure 9, Figure 10 and Figure 11. Figure 9 and Figure 10 are scatterplot matrix graphs that show correlations between selected stiffness-related and viscosity-related properties, respectively. Figure 11 is a scatterplot matrix table presented in the form of a heatmap that shows Pearson′s correlation coefficients between all pairs of measured properties including the glass transition temperature.

Figure 9 illustrates that the stiffness-related properties were directly proportional to each other for all length scales (macro-, micro- and nano) and for both quasi-static and dynamic properties (*G*′, *E*_IT_ and *H*_IT_). All correlations were reasonably linear, which was in perfect agreement with the theoretical prediction (Equation (4)). As expected, the biggest deviations from the linearity were observed between the most different properties, i.e., between the elastic moduli from dynamic experiments (*G*′) and hardness from quasi-static experiments (*H*_IT_). Nevertheless, all correlations (off-diagonal elements in Figure 9) were clearly visible and strong (the strength of correlations is quantified in Figure 11).

Figure 10 documents that the viscosity-related properties exhibited strong mutual correlations as well. Some pairs of viscosity-related properties were not directly proportional, but this was not in disagreement with theory (Equations (1)–(4)), which predicts positive linear correlations only for the stiffness-related properties. All trends in Figure 10 were quite logical and held for all length scales, which confirmed the reliability and reproducibility of our measurements. For example, a strong positive correlation among values of G″ (measured by dynamic experiments in macro- and nanoscale) and *C*_IT_ (measured by quasi-static experiments in micro- and nanoscale) proved that the samples with a higher contribution of viscosity (high G″) exhibited higher cold flow under long-term load (high *C*_IT_). Additionally, a strong negative correlation between the values of *n*_IT_ and *C*_IT_ (from both micro- and nanoindentation) evidenced that highly elastic materials (high *η*_IT_) did not tend to flow under loading (low *C*_IT_) and *vice versa*. A similar negative correlation was observed between *η*_IT_ and *G*″, where the highly elastic materials (high *η*_IT_) were not viscous (low *G*″) and *vice versa*.

Figure 11 summarizes and quantifies correlations between all measured properties. The strength of correlations is given by Pearson′s correlation coefficients *r* and emphasized by color—the strongest linear positive correlations (*r* close to +1) are marked black and the strongest linear negative correlations (*r* close to -1) are marked red. The correlation matrix is symmetric with respect to the main diagonal, which contains values equal to 1 (autocorrelations). Around the main diagonal, several distinct groups of strong positive linear correlations could be recognized. The black rectangular region in the upper left corner (from DMA/*G′* to NHI/*H*_IT_) evidences the very strong correlations between all stiffness related properties (all Pearson′s *r* > 0.9). The black rectangular region in the middle of the table (from DMA/*G*″ to NHI/*C*_IT_) proves the strong correlations between *G*″ (dynamic property representing viscous flow in general) and *C*_IT_ (quasi-static property representing cold flow under long-term load). The two small black rectangular regions in the lower right corner document very strong linear correlations between equivalent properties from macro- and nanoscale dynamic experiments (DMA/tanδ and NHI/tanδ) and from micro- and nanoscale quasi-static experiments (MHI/*η*_IT_ and NHI/*η*_IT_). The red rectangular regions in the lower left and upper right corner show medium negative correlations (*r* < −0.5) between four viscosity-related properties (DMA/tanδ, NHI/tanδ, MHI/*η*_IT_ and NHI/*η*_IT_) and all stiffness-related properties (from DMA/G′ to NHI/*H*_IT_). This corresponded to the sample behavior visualized in Figure 4, Figure 5, Figure 6, Figure 7 and Figure 8: Elastic work of indentation (*η*_IT_; Figure 5d) showed the highest values for rubbery samples with the lowest values of *T*_g_ (S05–S10), which were much softer than the remaining glassy polymers as documented by the values of *H*_IT_, *E*_IT_, and *G*′ at all length scales (Figure 5a,b, Figure 7a, and Figure 8). Damping factors (tanδ = *G*″/*G′*; Figure 7d) indicated the transition from glassy to rubbery state and reached the highest values for the samples with a high contribution of *G*″ (S03–S08). These samples had *T*_g_ close to the temperature of measurement and, as a result, their stiffness steeply decreased as proved by both quasi-static measurements (Figure 5a,b; values of *E*_IT_ and *H*_IT_) and dynamic measurements (Figure 7a,c; values of *G*′ and |G*|). The red rectangular regions in the bottom and on the right show strong negative correlations between the elastic part of the indentation work (MHI/*η*_IT_ and NHI/*η*_IT_) and parameters related to viscous flow (DMA/*T*_g_, DMA/*G″*, NHI/*G″*, MHI/*C*_IT_, and NHI/*C*_IT_). This confirmed that the rubbery samples with the highest elasticity exhibited low glass transition temperatures (strong negative correlation *η*_IT_—*T*_g_) and relatively low contribution of viscous flow (negative correlations with *G*″ and *C*_IT_).

We conclude that statistical analysis summarized in the form of a correlation matrix table (Figure 11) confirmed quantitatively all observed trends (Figure 9 and Figure 10). Firstly, we proved the strong positive linear correlations between all stiffness-related properties at all length scales, in agreement theoretical prediction (Equations (1)–(4)). Secondly, we evidenced numerous additional correlations between both stiffness- and viscosity-related properties, which were in agreement with general trends and behavior of the polymer networks with increasing *T*_g_. Moreover, we managed to prove that the correlations that were strong (high Peason′s correlation coefficients *r* in Figure 11) were also statistically significant (the low *p*-values listed in Supplementary Materials).

### 5.3. Small Differences among Mechanical Properties at Different Length Scales

Mechanical properties at all length scales showed not only analogous trends (Figure 5, Figure 6, Figure 7 and Figure 8) and strong correlations (Figure 9, Figure 10 and Figure 11), but also similar absolute values. The great majority of the calculated ratios between four corresponding quasi-static properties (NHI/*E*_IT_:MHI/*E*_IT_, NHI/*H*_IT_:MHI/*H*_IT_, NHI/*C*_IT_:MHI/*C*_IT_, and NHI/*E*_IT_:MHI/*E*_IT_) and three corresponding dynamic properties (NHI/*G*′:DMA/*G*′, NHI/*G*″:DMA/*G*″, and NHI/tanδ:DMA/tanδ) were within interval 0.5–1.5, which means that most of the values did not differ by more than 50% (the complete data are in Supplementary Materials). This was a surprisingly good result considering that we characterized an unusually broad range of materials (from very hard and brittle to very soft and elastic) using four independent methods (DMA, quasi-static MHI, quasi-static NHI, and dynamic NHI) at three different scales (macro-, micro- and nano).

The observed differences among mechanical properties at different length scales were comparable or even lower than in previous studies, which showed that the values of macro-, micro- and nanomechanical properties of polymer samples may differ significantly. Fasce et al. [56] characterized nitrogen ion irradiated UHMWPE surfaces by both MHI and NHI, and observed a steep increase in modulus and hardness between nanoindentation and microindentation range—the reduced modulus changed by as much as 50%. An even higher difference was reported by Kotsilkova et al. [57], who measured mechanical properties of poly(methyl methacrylate)/graphene thin films using small-punch testing (SPT), instrumented nanoindentation (NHI) and atomic force microscopy (AFM)—the surface elastic moduli obtained from AFM and NHI were quite comparable, but more than one order of magnitude higher than the bulk elastic moduli obtained from SPT. Ilie and Hickel [48] characterized various dental composites (crosslinked polymer resins with inorganic fillers) before and after aging—macromechanical tensile moduli were up to 2.5× lower than *E*_IT_ from quasi-static MHI measuremens and 2.1× lower than *E*′ from dynamic NHI measurements. Hardiman et al. [36] performed a detailed study of a stiff commercial epoxy resin and compared macroscopic tensile modulus with nanoindentation modulus—if the nanoindentation modulus was calculated in a standard way (i.e., in terms of Oliver and Pharr theory without additional sample-specific), it was ca. 1.4× higher than the macroscopic modulus.

In general, the differences between macro-, micro- and nanomechanical properties of polymers occur either due to core–shell effects (a real difference between surface and bulk properties of the investigated system) or due to complex elasto-visco-plastic behavior of polymer materials (which results in various phenomena, such as indentation-size effect [58], creep during loading and/or unloading [38], sink-in and pile-up effects [36], and nose effect [30]). A detailed review of Diez-Pascual et al. [18] confirmed that direct comparison of macroscopic properties with micro/nanoindentation results is quite challenging due to specific principle and geometry of indentation experiments, which can lead to all above-listed effects and dependence of final results on experimental conditions. The review contains many references to previous studies where the macroscale properties and indentation results were in fair agreement, but also references where a significant disparity was observed.

In our case, we strived to minimize the differences between macro-, micro- and nanomechanical properties in multiple ways. Foremost, we prepared a set of very homogeneous materials in order to eliminate core–shell effects. Additionally, the crosslinking density was modified just by changing the ratio of the components so that we did not introduce into our systems any new materials with different *E*/*Y* ratio that could break the expected linear correlations among stiffness-related properties. Finally, we used as close experimental parameters as possible (similar loading, unloading and hold time in quasi-static experiments and the same oscillation frequency in dynamic experiments). The change in experimental parameters would probably lead to different results. It is therefore recommended to use corresponding test parameters as close as possible for all test methods. Since polymers have temperature-dependent mechanical properties, it is also important to perform the measurements at the same temperature, especially on samples that have *T*_g_ close to the ambient temperature.

### 5.4. Small Difference between Indentation Hardness and Universal Hardness

In quasi-static MHI and NHI experiments, the stiffness-related properties (MHI/*H*_IT_, MHI/*E*_IT_, NHI/*H*_IT_, and NHI/*E*_IT_) were evaluated in terms of Oliver and Pharr’s theory [2,29]. This approach is nowadays incorporated in most commercial instruments and extensively used for the analysis of quasi-static indentation data including polymer systems [7,18]. However, the O&P theory was developed for elasto-plastic materials, but our samples changed from elasto-plastic, through elasto-visco-plastic to completely elastic (Figure 1, Figure 3 and Figure 4).

**Figure 12:**
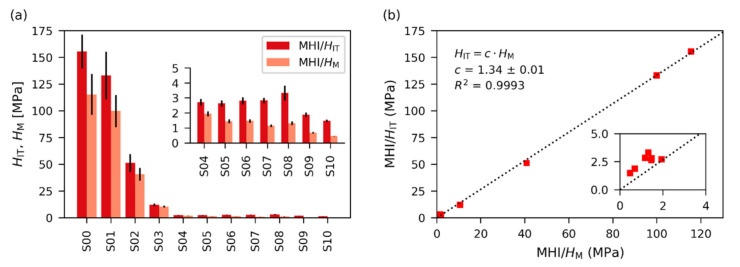
Results of quasi-static microindentation hardness testing: Difference between the values of indentation hardness (MHI/*H*_IT_) and Martens hardness (MHI/*H*_M_). Left figure (**a**) shows the difference between absolute values and right figure (**b**) shows the correlation between the two quantities. Error bars in left figure represent standard deviations.

In order to verify if the O&P theory was a good approximation in our case, we compared two quantities from quasi-static MHI data: Martens hardness (MHI/*H*_M_; calculated directly from experimental data) and indentation hardness (MHI/*H*_IT_; calculated in terms of O&P theory) [7,11,58]. Both quantities showed the same trends (Figure 12), which confirmed that the O&P theory is a reasonable approximation for our set of polymers in the whole range of crosslinking densities. The fact that MHI/*H*_IT_ values were higher than MHI/*H*_M_ (Figure 12a) was in agreement with the definition of both quantities (*H*_IT_ = *F*/*A*_p_ and *H*_M_ = *F*/*A*_d_, where *A*_p_ and *A*_d_ are projected and developed area of the indent, respectively [11]; this leads to *H*_IT_/*H*_M_ = *A*_d_/*A*_p_ ≈ 1.079 for Vickers microindentation as justified in the Supplementary Materials). Moreover, O&P theory assumes a sink-in effect, which further decreases the value of *A*_p_ [2]). The correlation between the MHI/*H*_M_ and MHI/*H*_IT_ was strong (Figure 12b; *R*^2^ = 0.9993) and MHI/*H*_M_ correlated well also with other stiffness-related properties at all length scales, as evidenced in Figure 11.

### 5.5. Limitations of Approximate Relations Used in This Study

The linear relation among stiffness-related properties (Equations (1)–(4)) of polymer systems is just an approximation, although it works surprisingly well for many polymer systems, including the series of crosslinked polymers studied in this work. From a historical perspective, the widely used Tabor′s relation (*H*/*Y* = 3, Equation (1); [1]) was derived for rigid-perfectly plastic materials; within this simplified model, the relation between *H* and *Y* is linear. In the next step, various researchers gradually developed models for elastic-perfectly plastic materials (*H*/*Y* = *f*(*E*/*Y*), where *f* is a function of *E*/*Y* ratio [59,60,61,62]); within these expanded cavity models (ECM) the linearity between *H* and *Y* holds only for constant *E*/*Y* ratio. More recent studies [63,64] further extended the ECM models to cover also materials exhibiting strain-hardening, such as elastic linear-hardening materials and elastic power-law hardening materials. Apart from complex theoretical derivations and considerations, all ECM-based models are in agreement that *H*/*Y* ratio increases with increasing *E*/*Y* ratio. Nonetheless, for common polymer materials, the *H*/Y ratio usually ranges from 2 to 3 and does not change too much (because the *E*/*Y* ratio for a given polymer system is frequently quite constant as exemplified in the above-discussed studies [12,32,33]) and this leads us back to the original Tabor′s relation. From the experimental point of view, Balta-Calleja and Kilian [65] studied a large set of polyethylene samples with various morphologies and crystallinities and showed that the *H*-*Y* relation was almost perfectly linear, while the *H*-*E* relation slightly deviated from linearity (*H* = *aE^b^*, but the exponent *b* was not too different from 1). Similarly, Lesah-Khosh [66] observed certain deviations from *H*-*Y*-*E* linear correlations in isotactic polypropylene samples. Many other studies confirmed fair linear correlations between indentation hardness and other stiffness-related properties [32,47,67,68,69,70,71]. We conclude that for many polymer systems the deviations from linearity in *H*-*Y*-*E* relations are not critical.

Even if the correlations among stiffness-related properties are linear as discussed in the previous paragraph, the proportionality constants (i.e., the constants in Equations (1)–(4)) can vary for different polymer systems. The Tabor′s ratio *H*/*Y* ≈ 3 (Equation (1)) is a surprisingly good approximation in most cases, although the proportionality constant is below the theoretical value and ranges from 2 to 3 [10]. The Struik′s ratio *E*/*Y* ≈ 30 (Equation (2)) fluctuates much more as a function of selected polymer systems and experimental conditions, which results also in variations of *E*/*H* ≈ 10 (Equation (4)). The approximate linear *E*-*Y-H* correlations for polymer systems were noticed by researchers even before Struik′s work [41] that justified the ratio *E*/*Y* ≈ 30. Two independent groups of authors [65,72,73] studied large sets of polyethylene samples with various morphologies and crystallinities and found strong linear *H*-*Y* correlation (*H*/*Y* ≈ 2.5) and roughly linear *H*-*E* correlation (*H* = *aE^b^*, where *b* ≈ 1.4). Moreover, Lorenzo et al. [73] introduced parameter 100*Y*/*E*, which changed from 8.5 (*E*/*Y* ≈ 12; branched polymers with low crystallinity) to 2.5 (*E*/*Y* ≈ 40; linear polymers with high crystallinity). These results indicated that a significant change in the polymer system could influence the *E*/*Y* ratio significantly. Later Flores et al. [68] confirmed linear *E*-*Y*-*H* correlations for both chain-extended and chain-folded polyethylenes; the observed ratios were very close to theoretically predicted values (*H*/*Y* ≈ 3, *E*/*Y* ≈ 30, and *E*/*H* ≈ 30) on condition that the values of *E* and *Y* were taken from tensile experiments. Gimenez et al. [69] found linear *E*-*Y*-*H* correlations for a large set of binary and ternary blends composed of ethylene–vinyl alcohol copolymer, amorphous polyamide, and polyamide-containing ionomer (*H*/*Y* ≈ 2, *E*/*Y* ≈ 37, and *E*/*H* ≈ 20). A few later studies compared *H*_IT_ and *E*_IT_ from instrumented microindentation experiments. Ostafinska et al. studied PLA/PCL blends [32,33] and found *E*_IT_/*H*_IT_ ≈ 15, which somewhat differed from the theoretical prediction (*E*/*H* ≈ 10), but the linear correlation between indentation modulus and hardness was clear and strong. Slouf et al. [12] characterized isotactic polypropylenes with various crystallinities and various content of α, β, and γ crystalline modifications and found *E*_IT_/*H*_IT_ ≈ 11 (quite close to the theoretical prediction). We conclude that the values of proportionality constants among *H*, *Y*, and *E* fluctuate for different systems and different experimental conditions, but the direct *H*-*Y*-*E* proportionality usually holds quite well, as confirmed by the above-cited studies and also by the results of this work. 

The increase in the stiffness-related properties with *T*_g_ (Equation (5)) for amorphous crosslinked polymers is a general rule and well-known trend, but it is worth re-emphasizing that it holds just within a given set of similar samples. In the systems of crosslinked polymers, in which the authors changed not only the crosslinking density, but also morphology, components and/or fillers of the crosslinked resin, the correlations between stiffness-related properties and glass transition temperature were very weak or disappeared completely [46,74]. In our series of homogeneous crosslinked epoxy resins, we changed crosslinking density (simultaneously with *T*_g_) by just varying the ratio of the components and so this problem was avoided. The reasons why the relationship between stiffness-related properties and *T*_g_ was not linear in the whole range is discussed briefly above (Section 5.2) and in more detail in the Supplementary Material.

### 5.6. Comparison with Previous Results

As mentioned in the Introduction, the number of studies comparing mechanical properties of polymers at all three length scales (macro-, micro-, and nano) is rather low. In the field of crosslinked polymers, the typical characterization method is DMA, while indentation methods are employed scarcely. The only notable exception is macroscopic Shore hardness that is often applied for characterization of elastic crosslinked rubbers; the correlations between Shore hardness and macroscale elastic moduli are frequently quite strong [75,76]. Moreover, the authors usually investigate either stiff resins (i.e., thermosetting resins and/or vitrified networks below their *T*_g_ [45,46,77]) or soft rubbers (i.e., elastic networks above their *T*_g_ [74,75,76]). The correlations between *T*_g_ and stiffness-related properties of those systems were usually weak, as the authors typically compared systems with different chemical compositions [46] and/or different amounts of various fillers [76]. Consequently, we did not find any study comparing macro-, micro-, and nanomechanical properties of homogeneous crosslinked polymers with the same components and increasing crosslinking density, whose properties change gradually from very hard and stiff to very soft and elastic (Figure 4). From this point of view, our work is original and, as a result, we could compare the observed trends and correlations only with the theoretical predictions and/or studies with samples exhibiting a narrower range of properties.

This work documented that the stiffness-related properties at all length scales increased monotonously with crosslinking density and glass transition temperature of highly homogeneous epoxy resin networks formed by common, well-established and well-defined commercial components (Jeffamine D400, Jeffamine D2000, and DGEBA). The prepared polymer networks were not only homogeneous, but also chemically similar as we changed the crosslinking density only by the variation of the ratio of the short and long elastic amino-functional chains (Jeffamine D400 and Jeffamine D2000). For these homogeneous and chemically similar systems, we found strong correlations among all corresponding macro-, micro- and nanomechanical properties. The available literature suggested that this was the first study characterizing the macro-, micro- and nanomechanical properties of crosslinked polymers with such a broad range of mechanical behavior. In the future, it would be worth verifying if such clear trends and strong correlations hold also for other types of polymer networks.

## 6. Conclusions

The aim of this work was to compare the macro-, micro- and nanomechanical behavior of crosslinked polymers. We prepared a set of chemically similar, highly homogeneous epoxy resin networks, whose mechanical properties at room temperature changed gradually with changing ratio of macro-comonomers from very hard and stiff (glassy state, frozen rubber), through semi-hard and ductile (glass transition near room temperature), to soft and elastic (rubbery). The elastic moduli at room temperature changed in an unusually broad range from 4 GPa to 0.006 GPa. The mechanical properties were characterized in the macroscale (dynamic mechanical analysis; DMA), microscale (quasi-static microindentation hardness testing; MHI) and nanoscale (quasi-static and dynamic nanoindentation hardness testing; NHI). The main results can be summarized as follows:

1. All three test methods (DMA, MHI and NHI) could be successfully applied to the characterization of the crosslinked polymer samples. The changes of the mechanical behavior and stiff–ductile–elastic transitions could be reliably detected not only by the dynamic methods (DMA and dynamic NHI), but also by the quasi-static methods (quasi-static MHI and NHI).
2. The stiffness-related properties (i.e., storage moduli, indentation moduli and indentation hardness at all length scales) showed strong and statistically significant correlations (all Pearson′s correlation coefficients *r* > 0.9 and corresponding *p*-values < 0.001). Moreover, the relations among all stiffness-related properties were approximately linear, in agreement with a theoretical prediction.
3. The viscosity-related properties (i.e., loss moduli, damping factors, indentation creep and elastic work of indentation at all length scales) yielded useful additional information about stiff-ductile-elastic transitions, although their mutual correlations were more complex in comparison with the stiffness-related properties: Loss moduli, damping factors and indentation creep showed one maximum for ductile samples with intermediate crosslinking density (the deformation of ductile polymers exhibited the highest contribution of viscosity), while elastic work of indentation showed two maxima for the hardest samples in the glassy state (acting as *enthalpic springs*) and the softest samples in the rubbery state (elastic rubbers, acting as *entropic springs*).
4. The similar values and trends of the corresponding macro-, micro- and nanomechanical properties (such as storage modulus from DMA and dynamic NHI, indentation hardness from quasi-static MHI and NHI, etc.) confirmed that micro- and nanoindentation are relevant methods for characterization of polymer materials with a broad range of properties. Both quasi-static and dynamic indentation methods can be employed as an alternative to traditional and well-established DMA analysis of crosslinked polymers.
5. The strong correlations among the corresponding macro-, micro- and nanomechanical properties confirmed the reliability of our measurements and pointed out that additional indentation results, such as indentation creep or elastic work of indentation are useful properties for characterization of polymer materials, despite being often neglected.

## Figures and Tables

**Table 1:** List of prepared samples.

Sample ID	Molar Ratio ^1^ of Components: DGEBA:D400:D2000	*T*_g_^2^ (°C)
S00	2:1:00:0.00	59.5
S01	2:0.98:0.02	55.0
S02	2:0.95:0.05	49.0
S03	2:0.92:0.08	44.7
S04	2:0.85:0.15	32.5
S05	2:0.80:0.20	27.8
S06	2:0.78:0.22	26.0
S07	2:0.70:0.30	16.9
S08	2:0.65:0.35	12.7
S09	2:0.30:0.70	−11.3
S10	2:0.00:1.00	−23.2

^1^ Only stoichiometric formulations were prepared; the stoichiometry being defined by the molar ratio of functional groups r = (amino-H)/(epoxy) = 1. As the amino-components D400 and D2000 were H-tetrafunctional, and the epoxy component DGEBA contained two epoxy groups, the molecular ratios were 2 DGEBA:1 diamine. **^2^** The values of the glass transition temperature (*T*_g_) were determined in the macro-scale dynamic-mechanical thermal analysis tests (carried out at 5 Hz) as the temperatures of the peak maximum of the temperature-dependent loss factor (tanδ). The plots of tanδ can be found in the Supplementary Materials.

## Supplementary Materials

The following are available online at https://www.mdpi.com/2073-4360/12/12/2951/s1, Supplementary text with the following contents: (1) Stiffness-related properties of amorphous and crosslinked polymers, (2) Detailed description and specific features of epoxy resins used in this work, (3) Tables with all measured macro-, micro- and nanomechanical properties, (4) Statistical significance of correlations among mechanical properties, (5) Differences among values of macro-, micro- and nanomechanical properties, and (6) Definitions and different values of *H*_IT_ and *H*_M_.
