# Supplementary material for: Macro-, Micro- and Nanomechanical Characterization of Crosslinked Polymers with Very Broad Range of Mechanical Properties
Source: Polymers (Basel). 2020 Dec 10;12(12):2951. doi: 10.3390/polym12122951 (PMC7763541; doi:10.3390/polym12122951)
Supplement: Supplementary file 1 [file polymers-12-02951-s001.pdf]

Macro-, micro- and nanomechanical characterization of crosslinked polymers with very broad range of mechanical properties

Supplementary materials

Miroslav Slouf, Beata Strachota, Adam Strachota, Veronika Gajdosova, Vendulka Bertschova, Jiri Nohava

Contents

1 Stiffness-related properties of amorphous and crosslinked polymers vs. T_g 2
1.1 Amorphous polymers 2
1.1.1 Amorphous polymers above their T_g 2
1.1.2 Amorphous polymers below their T_g 2
1.2 Crosslinked polymers 3
1.2.1 Crosslinked polymers above their T_g 3
1.2.2 Crosslinked polymers below their T_g 3
1.3 Different deformation behavior of polymer networks above and below T_g 4
2 Detailed description and specific features of epoxy resins used in this work 7
2.1 Synthesis and structure 7
2.2 Homogeneity of crosslinking 7
2.3 Thermomechanical analysis (DMA) 8
2.3.1 Auto-strain function applied in DMA 8
2.3.2 Glass transition temperatures of the polymer networks 9
2.3.3 Crosslinking density analysis obtained from DMA 9
3 Tables with all measured of macro-, micro- and nanomechanical properties 12
4 Statistical significance of correlations among mechanical properties 13
5 Differences among values of macro-, micro- and nanomechanical properties 14
6 Definitions and different values of H_{IT} and H_M 16
References 17

1 Stiffness-related properties of amorphous and crosslinked polymers vs. T_g

Stiffness-related properties (i.e. hardness, *H*, yield stress, *Y*, elastic modulus, *E,* and shear modulus, *G*) of amorphous and crosslinked polymers are closely connected with the glass transition temperature (T_g), which is influenced by the crosslinking density (characterized by the average molar mass between crosslinks, M_c). The combination of the fact that stiffness-related properties of polymer systems are approximately proportional to each other (Eq. 4 in the main text) and the semi-empirical fact that the stiffness-related properties tend to increase with T_g can be written as follows (Eq. 5 in the main text):

$$H \approx 3Y \approx E/10 \approx [2G(1+\nu)]/10 \approx f(T_g) \quad \text{(S1)}$$

where $f(T_g)$ is a function growing with T_g. The exact shape of the function depends on the investigated system, but for some specific types of amorphous and/or crosslinked polymers its approximate shape is known as exemplified below.

1.1 Amorphous polymers

1.1.1 Amorphous polymers above their T_g

Non-crosslinked amorphous polymers above their glass transition temperature are viscous liquids, even if their viscosity can be quite high. The study of their stiffness-related properties is of little practical importance, as their dominating feature is viscous flow. The production and applications of amorphous polymers with T_g below ambient temperature are limited. One of a few real-life examples is atactic polypropylene (amorphous polymer; $T_g \approx$ -10 °C), which a soft, tacky and rubber-like material at laboratory temperature; it is employed mostly as sealant or additive to bitumen [Rishina 2019].

1.1.2 Amorphous polymers below their T_g

Non-crosslinked amorphous polymers below their glass transition temperature are usually stiff and brittle. Their stiffness-related properties at laboratory temperature depend on T_g strongly. Some of them (such as polystyrene or polyethylene terephthalate) belong among the most common, commodity polymers produced in megatons per year. Gedde [Gedde 1995] summarizes that T_g of amorphous polymers increases mainly with increasing attractive forces between polymer chains (i.e. with cohesive energy density), with the rigidity of the main chain and/or side chains (i.e. with decreasing internal mobility of the chains) and with the degree of crosslinking (chemical crosslinks fix the polymer chains and decrease the free volume). Fakirov [Fakirov 1999] found a semi-empirical linear relation between T_g and hardness of non-crosslinked, glassy amorphous polymers (Eq. S2a; *k* and *C* are constants). Combination of this relation with Eq. S1 indicates that all stiffness-related properties of amorphous polymers increase with glass transition temperature (Eq. S2b):

$$H = k \cdot T_g + C \quad \text{(S2a)}$$

$$H \approx 3Y \approx E/10 \approx [2G(1+\nu)]/10 \approx k \cdot T_g + C \quad \text{(S2b)}$$

Equation S2a was derived on the basis of Vickers microhardness data collected for fourteen common, commercially available amorphous homo- and copolymers, whose main chains contained only single C-C, C-O or C-N bonds [Fakirov 1999; Balta-Calleja 2000]. This set yielded quite good linear relationship (Eq. S2a with *k* = 1.97 MPa/K, *C* = -571 MPa, and correlation coefficient 0.96 [Fakirov 1999; Fakirov 2000]). Later it was found that some polymers may deviate from the trend, such as polycarbonate

(negative deviation, ref. [Balta-Calleja 2004]) or amorphous polylactide (positive deviation, ref. [Ostafinska 2015]). Nevertheless, the general trend given by Eq. S2a-b (i.e. approximately linear increase in H and other stiffness-related properties with T_g) holds for amorphous polymers quite well [Flores 2009].

1.2 Crosslinked polymers

For crosslinked polymers the relation between stiffness-related properties and glass transition temperature is less straightforward that in the case of amorphous polymers discussed above. From the point of theory, we must consider two distinct cases: crosslinked polymers above T_g (elastic rubbers) and above T_g (thermosetting resins or vitrified networks).

1.2.1 Crosslinked polymers above their T_g

For crosslinked polymers above T_g, the theory of rubber elasticity yields a relation between elastic modulus and crosslinking density characterized by the number of elastically active chains, N, or average molar mass between crosslinks, M_c (Eq. S3a; where k is Boltzmann constant, R is gas constant, T is temperature of measurement and ρ is density of the rubber [Ward 2004]). Combination of this relation with Eq. S1 indicates that stiffness-related properties of elastic rubbers increase with increasing crosslinking density (Eq. 7b):

$$E = NkT = \rho RT/M_c \qquad \text{(S3a)}$$

$$H \approx 3Y \approx E/10 \approx [2G(1+\nu)]/10 \approx f(T_g) \approx \rho RT/10M_c \qquad \text{(S3b)}$$

It is worth re-emphasizing that Eq. S3a is rather approximate, but its validity (for chemically similar crosslinked polymers) has been proved by many previous authors [Yin 2013, Ramsdale-Capper 2018, Slouf 2018, Kupka 2019]. Equation S3b gives no direct information about T_g, but a general qualitative trend (for chemically similar polymers) is that higher crosslinking density means not only higher stiffness, but also higher T_g due to limited chain mobility.

1.2.2 Crosslinked polymers below their T_g

Crosslinked polymers below T_g (vitrified networks or thermosetting resins) are in the glassy state. The theory of polymers in the glassy state [Gedde 1995, Ward 2004] relates the T_g and M_c ($T_g \approx C_9 + C_{10}/M_c$, where M_c was defined above and C_9 with C_{10} are constants [Gedde 1995]). In the region of low deformations, where strain hardening does not play an important role, both non-crosslinked and crosslinked glassy polymers exhibit similar mechanical behavior [Ward 2004]. Therefore, also for vitrified networks the stiffness-related properties tend to increase with glass transition temperature and crosslinking density (according to Eq. S1).

The increase in stiffness-related properties with T_g of the crosslinked polymers is not linear in the whole range. The reason of the non-linear behavior is illustrated in Fig. S1: the same shift in T_g does not always lead to the same shift in DMA/G'. In our case, there was a clear difference between the first group of samples with T_g above laboratory temperature (red squares in Fig. S1a–b) and the second group of samples with T_g close to and below laboratory temperature (violet circles in Fig. S1a–b). Moreover, even the linear correlations between T_g and DMA/G' observed in the two distinct groups of samples (Fig. S1b) were just approximate. Fig. S1a suggests that the observed linear correlations in the two

distinct regions are partially a coincidence and for different system the deviations from linearity could be even higher.

We note that Fig. S1 shows the relation between T_g and DMA/G'. The shear modulus (DMA/G') represents just one of the stiffness-related properties determined in this work. Nevertheless, the analogous conclusions hold also for other stiffness-related properties in this study discussed in the main text (MHI/E_{IT}, MHI/H_{IT}, NHI/E_{IT}, NHI/H_{IT}, and NHI/G'). This is due to the approximately linear correlations between the stiffness-related properties of the polymer systems, which are both predicted by theory (Eq. S1 above; Eq. 1–5 in the main text) and confirmed by our results (see Discussion and Fig. 9 in the main text).

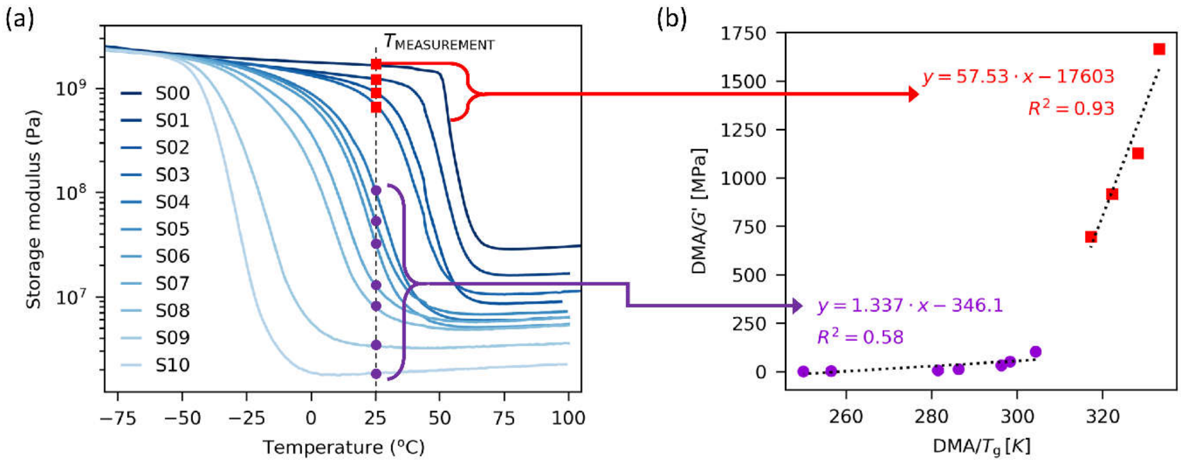

Figure S1. Illustration of the relation between storage modulus from dynamic mechanical analysis (DMA/G') and glass transition temperature (T_g). The left figure (a) shows the raw data from DMA experiments, while the right figure (b) shows correlation between DMA/G' and T_g. The samples with T_g above laboratory temperature (S00–S03 in Fig. S1a; red squares in Fig. S1b) exhibit high shear moduli (ca 10^9 Pa), which increase steeply as a function of T_g. The samples with T_g close to and below laboratory temperature (S04–S10 in Fig. S1a; violet circles in Fig. S1b) exhibit substantially lower shear moduli (ca 10^6–10^8 Pa), which increase moderately as a function of T_g.

1.3 Different deformation behavior of polymer networks above and below T_g

At this place we recapitulate some known theoretical aspects concerning the deformation behavior, elasticity and viscoelasticity of the studied materials. All the polymers tested in our study consisted of flexible polymer chains crosslinked by rigid multivalent structural segments.

In the crosslinked polymers measured below their T_g, all movements of the polymer chains are frozen. The frozen polymer chains cannot even change their conformations. This rigidity is due to energy barriers for conformational rotation. Consequently, the applied deformation causes just *slight changes of intermolecular distances*, which is connected with enthalpic contribution to the free energy of the system ($\Delta G = \Delta H - T\Delta S$, where ΔH and ΔS represent enthalpic and entropic changes, respectively). That is why the elastic response of the tested material in its glassy state can be referred to as *enthalpic-spring-type* (or just briefly as *enthalpic spring*) because it is driven by enthalpy changes. The enthalpy effects are generated by Van der Waals forces, which are responsible for inter-molecular attraction (as well as repulsion upon compression), and which are considerably strong between large macromolecules (this results in the high elastic moduli in the range of 10^9 Pa). It has been observed [Gedde 1995, Balta-Calleja 2000] that the energy needed for deformation (as well as T_g and stiffness-

related properties) increases with cohesive energy density (CED), a quantity providing an integrated measure of the secondary (non-covalent) intermolecular bonds in a material. This latter magnitude hence is a measure of the strength of the above-mentioned Van der Waals forces between constituent macromolecules (or in case of the studied networks – between the linear chain segments). In case of the glassy state of polymer networks, the behavior is nearly ideally elastic at small deformations, but plastic deformations can occur, if molecular segments are shifted along each other too far. Due to their curly shape caused by individual atoms, the shifted segments eventually lock in a new position and are held in it by strong mutual Van der Waals forces. Unless the overall force caused by the deformation is too high, the small 'locked' plastic deformation can persist.

The crosslinked polymers measured above their T_g contain flexible polymer chains, whose movement is restricted mostly by crosslinks. The flexible chains easily change their conformations (free change of dihedral angles in the repeat units) and thus (via combination of local conformations of the numerous conformationally active repeat units) they can assume different 'global shapes', ranging between fully uncoiled ('straight', extreme case) and ideally coiled. In case of the fully uncoiled (straight) shape, only one combination ('micro-state') of local conformations yields this 'macro-state'. In case of the ideally coiled (spherical) shape, very numerous combinations of local conformations of the chain (micro-states) lead to this same 'macro-state'. Thus, from the viewpoint of the statistic theory of entropy, the fully stretched shape of the chain has an entropy of 0, while the ideally coiled shape has a high entropy. Partly stretched global shapes can be realized by a smaller number of micro-states than the ideally coiled shape, and thus have a lower entropy, but still a higher one than the fully stretched shape. These differences in entropy cause differences in free energy and thus cause an elastic retraction force. Because of its phenomenological nature, this effect in rubbery polymers is called the *entropic spring*. The equation for the (Gibbs) free energy holds: $\Delta G = \Delta H - T\Delta S$, where in case of ideal rubbery materials $\Delta H = 0$. The rubbery elastic response to a given deformation also increases with increasing experiment temperature, as given by the term $T\Delta S$, as far as there is no chemical degradation or pyrolysis. A typical entropic spring in polymers causes much smaller energy changes upon sample deformation in comparison to the 'Van der Waals enthalpic spring' which acts above T_g . Hence, the rubbery networks below their T_g are soft (their typical moduli are in the range of 10^6 Pa). The Van der Waals forces between macromolecular segments of course persist also in the rubbery state, but deformation in this state occurs exclusively via conformational changes (coiling/uncoiling), rather than by change of distance between macromolecular segments (the latter would be much more energy consuming). In the perfect case, the rubbery state is ideally elastic, and the entropic spring achieves very fast and 100% retraction of the previously applied deformation. Deviations from ideal behavior can be caused by entanglements of longer polymer chains, which can be disentangled or newly formed, or by crosslink disconnection (mechanical damage) at high strains. Some small degree of viscosity (internal friction) is often observed and is caused by friction (collisions) of individual moving polymer chain segments during deformation and retraction. In such cases ΔH in the above equation is somewhat higher than 0, but non-directional, always positive, independently of deformation or retraction.

To sum-up, the glassy as well as the rubbery states of a polymer network exhibit highly elastic behavior, albeit via different mechanisms (enthalpic vs. entropic). However, a *different behavior*, a *highly viscoelastic* one is observed *in the glass transition region*. In the equation $\Delta G = \Delta H - T\Delta S$ for deformations in this region, both ΔH and ΔS are non-zero. The situation is somewhat similar like in case of the rubbery state, but additionally, the term ΔH causes non-directional frictional resistance to deformation ('viscosity effect'). This frictional term is caused by barriers to conformational rotations

which are non-negligible in the temperature range of the glass transition region, as well as by Van der Waals attraction of the occasional ‘partly frozen’ segments. The viscosity effect is strong in the transition region and it grows as the temperature approaches the glassy region. Nevertheless, the behavior in the transition region is not truly plastic (it cannot be compared to materials such as metallic lead at room temperature), because most of the frictional resistance is eventually overcome by the entropic spring after a certain relaxation time, except in temperature regions close to the truly glassy state. Residual plastic deformations (entanglements stabilized by friction effects and Van der Waals forces between frozen segments) can be expected to be the highest in the studied polymers, if they are in this specific thermal state.

2 Detailed description and specific features of epoxy resins used in this work

2.1 Synthesis and structure

Scheme S1 depicts the synthesis and structure of the epoxy networks tested as specimens in this work (common literature name: diglycidyl ether of bisphenol A cured with diamine terminated polypropylene oxide; IUPAC name: net-poly[(2,2-Bis(4-glycidyloxyphenyl)propane)-co-(polypropyleneoxide-α,ω-diamine)]; semi-trivial name: net-poly[(bisphenol A diglycidyl ether)-co-(polypropyleneoxide-α,ω-diamine)]). The structure consists of semi-rigid chains where the diaromatic epoxy component DGEBA alternates with N atoms. These semi-rigid chains are mutually connected by highly flexible polypropyleneoxide chains which are attached to the nitrogen atom. This molecularly blended structure of two types of chains yields a single glass transition temperature (T_g), which is different from the T_g values of the constituent chains.

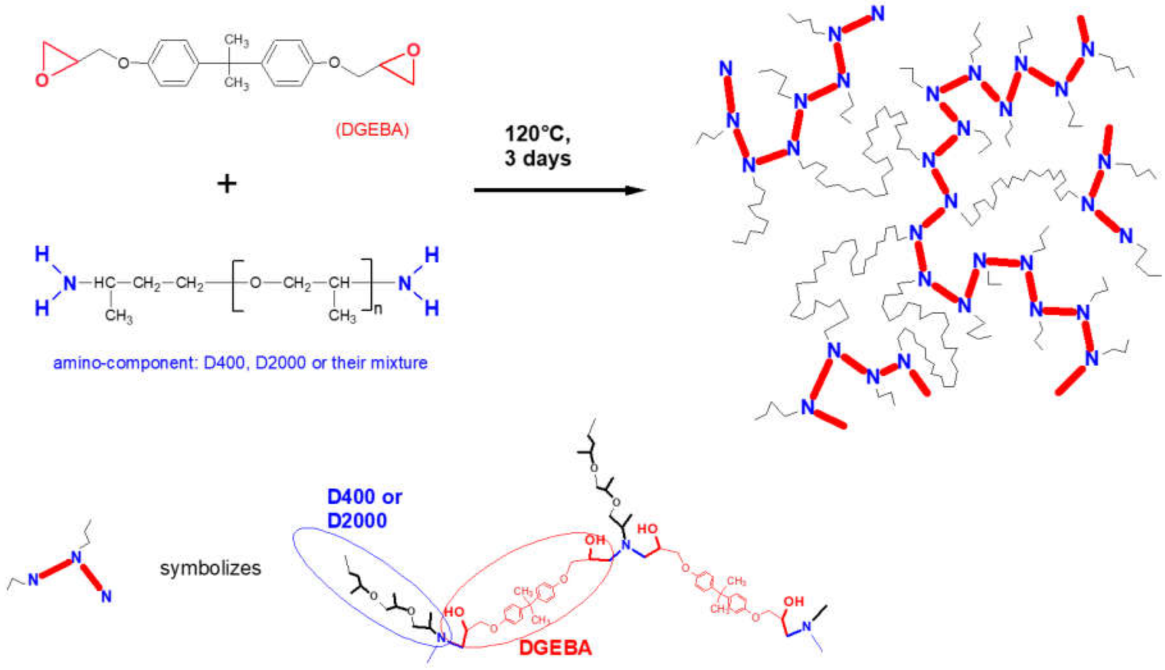

Scheme S1. Synthesis and structure of the tested epoxy networks.

2.2 Homogeneity of crosslinking

The homogeneity of crosslinking in the prepared epoxy networks was of great importance for the mechanical tests via indentation. Important is the behavior of the two diamine chains of highly different length during the epoxy cure, and their eventual tendency to phase separation. The homogeneity of this well-known epoxy system on the molecular scale was tested early on, in the 1980s. Generally, the nearly ideal homogeneity is supported by several literature findings and direct observations:

- Firstly, a visual check (as a rough method) shows good transparency and no opalescence, which means that eventual distinct heterogeneities must be smaller than ca. 100 nm.
- Secondly, finer-scale homogeneity studies were carried out in the literature, on networks with mixed chain lengths, like the ones used in this work. The studies were performed by small-angle X-ray scattering (SAXS) [Tan 1999] and by small-angle neutron scattering (SANS) [Wu

1986a; Wu 1986b]. The studies proved ideal homogeneity in the simpler network variety with one chain length [Wu 1986a], while an alternating block structure was observed for the bimodal networks [Wu 1986b; Tan 1999], with maximum size of nano-domains containing only one type of chains being around one nanometer, so that these networks also can be regarded as nearly ideally homogeneous, except at the smallest nanometer-scale.

- Thirdly, the tested bimodal networks display just one T_g, which confirms that the two network structures – wide mesh and narrow mesh – are mixed on the molecular level, and not forming separate domains [Gedde UW: Polymer Physics, Chapman and Hall, London 1995, p. 82]. The T_g values are namely highly different for eventual separate domains, as can be seen in Table 1 in main Manuscript for DGEBA-D400 and DGEBA-D2000.
- Finally, the samples were measured by four independent methods (DMA, quasi-static microindentation, quasi-static nanoindentation, and dynamic nanoindentation) in this work; for each method we employed a different piece of the prepared samples and the strong correlation between stiffness related properties at all length scales (including nanoscale) was achieved; this would not be possible if the homogeneity of the samples were bad. There were no anomalous fluctuations in the nanoindentation results of the bimodal networks.

Concerning the eventual effect of fluctuations in crosslink density on indentation results, it is important to note, that the minimal size of the indents (which could be estimated from the known penetration depths and hardness of the specimens) was ca 70 µm for microindentation and ca 30 µm for nanoindentation. This is many orders a larger size than the one of the heterogeneities in crosslink density. Therefore, the micro- and nanoindentation results obtained in this work confirmed that the samples were homogeneous in at the scale of tens of micrometers.

2.3 Thermomechanical analysis (DMA)

2.3.1 Auto-strain function applied in DMA

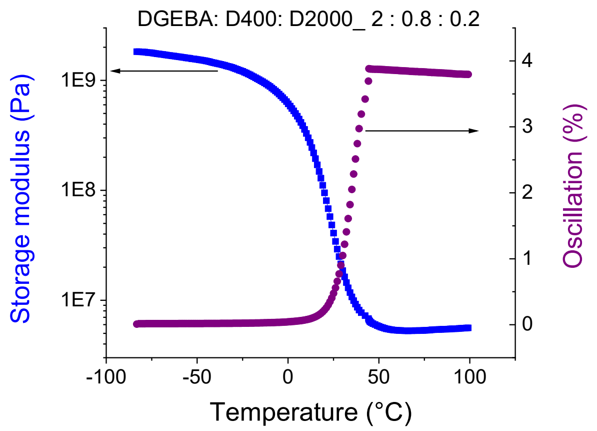

Figure S2. Storage modulus and applied oscillation strain as function of temperature for sample S05 (see Table 1 in the Experimental section of the main text for the description of all samples).

An oscillatory shear deformation at the constant frequency of 5 Hz and at the heating rate of 3°C/min was applied, and the temperature dependences of the storage shear modulus and loss modulus as well as of the loss factor (G’, G’’ and tan(delta), respectively) were recorded. The initial value of the oscillatory shear deformation was set at 0.01% and an *Auto-strain function* was applied, which upon

softening of the material (if torque value fell below 2000 μN·m) caused a gradual increase of the deformation amplitude, maximally up to the limit value of 4%, which was never exceeded (it was verified, that the linear deformation region in the studied samples extends at least up to 20%). The *Auto-Strain function* was used in order to obtain good quality data points in the rubbery region, where higher deformations are required. The temperature range of the analyses was typically from -90 to +100 °C. The example of the curve obtained with *Auto-strain function* is given in Fig. S2.

2.3.2 Glass transition temperatures of the polymer networks

The glass transition temperatures (T_g) of the studied polymers were determined by dynamic-mechanical thermal analysis (DMA): The T_g values were defined as the temperatures of the maxima of the curves of temperature-dependent loss factor tan delta (see **Figure S3**).

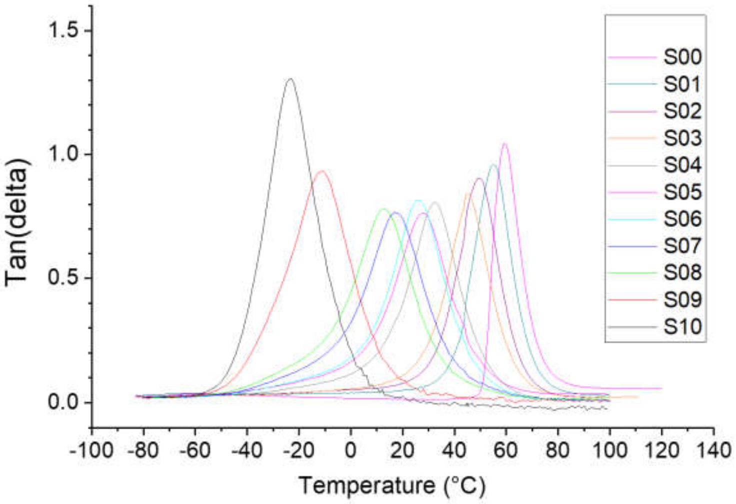

Figure S3. Dynamic-mechanical thermal analysis (DMA) of the studied polymers: temperature-dependent loss factor *tan delta*. The maxima of the curves were used to define the glass transition temperatures.

2.3.3 Crosslinking density analysis obtained from DMA

Crosslinking density was evaluated from the DMA measurements of the temperature-dependent storage shear moduli (Figure S1, or Figure 6 in the main Manuscript): The magnitude "concentration of the elastically active chains", *c*(EAC), was used to characterize the crosslink density, because this magnitude is most popular in the literature about rubbery networks. Its theoretical value also was calculated from sample volume and from the molar amount of the elastic chains (taken from the components amounts used for sample synthesis). The experimental value was calculated using the simple formula: $c(\text{EAC}) = G'/(R*T)$, where G' is a modulus value measured in the rubbery region, R is the gas constant and T is the temperature, at which the modulus was measured. The results are given in Table S1a–c.

Table S1a (1st part of the table)**.** Amounts of the reactant components for the synthesis of the tested polymer networks, calculation of the theoretical concentrations of elastically active chains from them, as well as calculation of experimental concentrations of elastically active chains from moduli in rubbery state and from the temperatures at which the moduli were measured.

Sample	DGEBA component*		D400 component*		D2000 component*	
ID	m (g)	n (mmol)	m (g)	n (mmol)	m (g)	n (mmol)
S00	1	2.935	0.6345	1.4675	0	0
S01	1	2.935	0.6218	1.4400	0.0578	0.0300
S02	1	2.935	0.6028	1.3950	0.1445	0.0725
S03	1	2.935	0.5838	1.3500	0.2313	0.1175
S04	1	2.935	0.5394	1.2475	0.4336	0.2200
S05	1	2.935	0.5076	1.1750	0.5781	0.2925
S06	1	2.935	0.4949	1.1450	0.6359	0.3225
S07	1	2.935	0.4442	1.0275	0.8672	0.4400
S08	1	2.935	0.4124	0.9550	1.0117	0.5125
S09	1	2.935	0.1904	0.4400	2.0234	1.0275
S10	1	2.935	0	0	2.8906	1.4675

* For checking stoichiometry, the functionalities of DGEBA (epoxy component, fn = 2) and of the amine components D400 and D2000 (fn = 4) must be considered.

Table S1b (2nd part of the table, continuation of Table S1a)**.** Amounts of the reactant components for the synthesis of the tested polymer networks, calculation of the theoretical concentrations of elastically active chains from them, as well as calculation of experimental concentrations of elastically active chains from moduli in rubbery state and from the temperatures at which the moduli were measured.

Sample ID	Sample mass (g)	Sample volume (mL) *	n(elast.chains) (mmol) **	c(EAC, theor.) (mol/L)
S00	1.6345	1.4859	1.4675	0.988
S01	1.6796	1.5269	1.4675	0.963
S02	1.7473	1.5885	1.4675	0.924
S03	1.8151	1.6501	1.4675	0.889
S04	1.9730	1.7936	1.4675	0.818
S05	2.0857	1.8961	1.4675	0.774
S06	2.1308	1.9371	1.4675	0.758
S07	2.3114	2.1013	1.4675	0.698
S08	2.4241	2.2037	1.4675	0.666
S09	3.2138	2.9216	1.4675	0.502
S10	3.8906	3.5369	1.4675	0.415

* The sample volume was calculated using the density of 1.10 g/mL for all the networks.
** Number of elastic chains means total number, i.e. D400 + D2000.

Table S1c (3rd part of the table, continuation of Table S1b)**.** Amounts of the reactant components for the synthesis of the tested polymer networks, calculation of the theoretical concentrations of elastically active chains from them, as well as calculation of experimental concentrations of elastically active chains from moduli in rubbery state and from the temperatures at which the moduli were measured.

Sample ID	G' (at selected T) (Pa)	T value (of G') (°C)	c(EAC, exp.) (mol/L) *	Percent of theory
S00	3.233E+07	120.1	9.889	1001.3
S01	1.521E+07	100.1	4.901	509.1
S02	9.000E+06	97.1	2.924	316.5
S03	1.125E+07	100.1	3.625	407.6
S04	5.970E+06	80.1	2.033	248.4
S05	6.950E+06	80.1	2.366	305.7
S06	5.200E+06	80.1	1.770	233.7
S07	5.950E+06	80.1	2.026	290.1
S08	5.040E+06	80.1	1.716	257.7
S09	3.300E+06	60.1	1.191	237.1
S10	1.920E+06	39.1	0.740	178.3

* *c*(EAC, experimental) was calculated using the formula: $c(\mathrm{EAC}) = G'/(\mathrm{R}*T)$, where *G'* is a modulus value measured in the rubbery region, R is the gas constant and *T* is the temperature, at which the modulus was measured.

3 Tables with all measured of macro-, micro- and nanomechanical properties

Table S2. Results of quasistatic microindentation (average ± standard deviation).

Sample ID	MHI/E_{IT} (MPa)	MHI/H_{IT} (MPa)	MHI/H_M (MPa)	MHI/C_{IT} (%)	η_{it} (%)
S00	3829 ± 377	155.48 ± 15.69	115.46 ± 19.07	14.5 ± 1.4	22.7 ± 1.6
S01	3362 ± 297	133.19 ± 22.20	99.90 ± 15.07	17.5 ± 2.7	22.0 ± 1.8
S02	1819 ± 135	51.38 ± 8.32	40.80 ± 5.98	37.3 ± 2.7	13.5 ± 0.9
S03	840 ± 51	12.33 ± 1.18	10.55 ± 0.97	55.3 ± 3.2	7.1 ± 0.2
S04	54 ± 7	2.73 ± 0.22	1.94 ± 0.17	39.0 ± 2.1	22.9 ± 1.2
S05	25 ± 3	2.64 ± 0.21	1.46 ± 0.13	18.2 ± 1.1	46.7 ± 1.5
S06	24 ± 2	2.82 ± 0.24	1.47 ± 0.12	13.7 ± 0.7	53.0 ± 1.4
S07	16 ± 1	2.85 ± 0.18	1.16 ± 0.09	5.5 ± 0.6	77.9 ± 1.6
S08	18 ± 2	3.34 ± 0.50	1.32 ± 0.13	5.1 ± 1.5	79.6 ± 5.3
S09	9 ± 1	1.89 ± 0.15	0.69 ± 0.06	2.2 ± 0.2	92.7 ± 0.8
S10	6 ± 0	1.49 ± 0.08	0.47 ± 0.02	1.6 ± 0.1	97.7 ± 0.3

Table S3. Results of quasistatic nanoindentation (average ± standard deviation).

Sample ID	MHI/E_{IT} (MPa)	MHI/H_{IT} (MPa)	MHI/C_{IT} (%)	η_{it} (%)
S00	3486.0 ± 291.8	147.17 ± 20.02	11.6 ± 1.8	25.8 ± 2.7
S01	3296.7 ± 168.1	132.11 ± 13.57	14.1 ± 1.5	24.4 ± 1.0
S02	2110.8 ± 154.1	77.72 ± 7.68	18.2 ± 2.5	21.0 ± 1.7
S03	777.8 ± 54.5	14.34 ± 1.41	48.3 ± 5.2	9.2 ± 0.7
S04	41.5 ± 1.8	2.20 ± 0.12	30.0 ± 0.9	26.8 ± 0.4
S05	19.0 ± 0.4	1.99 ± 0.04	12.2 ± 0.3	53.8 ± 0.5
S06	14.7 ± 0.7	1.87 ± 0.08	9.6 ± 0.3	61.8 ± 0.8
S07	14.1 ± 1.2	2.29 ± 0.09	6.1 ± 1.0	75.4 ± 3.9
S08	11.2 ± 0.4	2.06 ± 0.14	4.3 ± 0.3	83.8 ± 1.1
S09	5.9 ± 0.2	1.29 ± 0.05	3.1 ± 0.3	90.7 ± 1.2
S10	4.4 ± 0.2	1.08 ± 0.06	1.9 ± 0.1	96.2 ± 0.3

Table S4. Results of dynamic methods: DMA and quasistatic nanoindentation.

Sample ID	DMA/G' (MPa)	DMA/G'' (MPa)	DMA/tanδ ( )	DMA/T_g (C)	NHI/G' (MPa)	NHI/G'' (MPa)	NHI/tanδ ( )
S00	1667.2	24.0	0.014	60.0	1360.6	38.0	0.014
S01	1127.3	62.7	0.056	55.0	1355.3	46.7	0.056
S02	916.9	94.9	0.104	49.0	993.5	84.0	0.104
S03	696.2	109.9	0.158	44.0	421.5	95.2	0.158
S04	103.4	60.4	0.584	31.1	54.8	37.6	0.584
S05	52.5	38.4	0.732	25.1	17.9	14.5	0.732
S06	32.5	26.5	0.814	23.1	11.9	9.3	0.814
S07	12.7	7.4	0.58	13.1	7.4	3.6	0.580
S08	7.9	3.4	0.425	8.3	5.6	1.6	0.425
S09	3.4	0.2	0.058	-16.7	2.3	0.1	0.058
S10	1.8	0.0	0.009	-23.2	1.8	0.0	0.009

4 Statistical significance of correlations among mechanical properties

Figure S4 summarizes statistical significance of correlations among all measured macro-, micro- and nanomechanical properties (by means of *p*-values). It supplements Figure 11 in the main text, which displays the strength of the correlations (by means of Pearson's correlation coefficients *r*). The statistical evaluation of the results (including the brief description of Pearson's *r* and *p*-values) is described in the Experimental section of the main text. Briefly, *p*-value in represents probability that we would have obtained given or stronger correlation just by coincidence; the correlation is regarded as statistically significant if *p*-value > 0.05. The comparison of Figure 11 (main text) and Figure S4 (below) proves that the strong correlations were also statistically significant and *vice versa*.

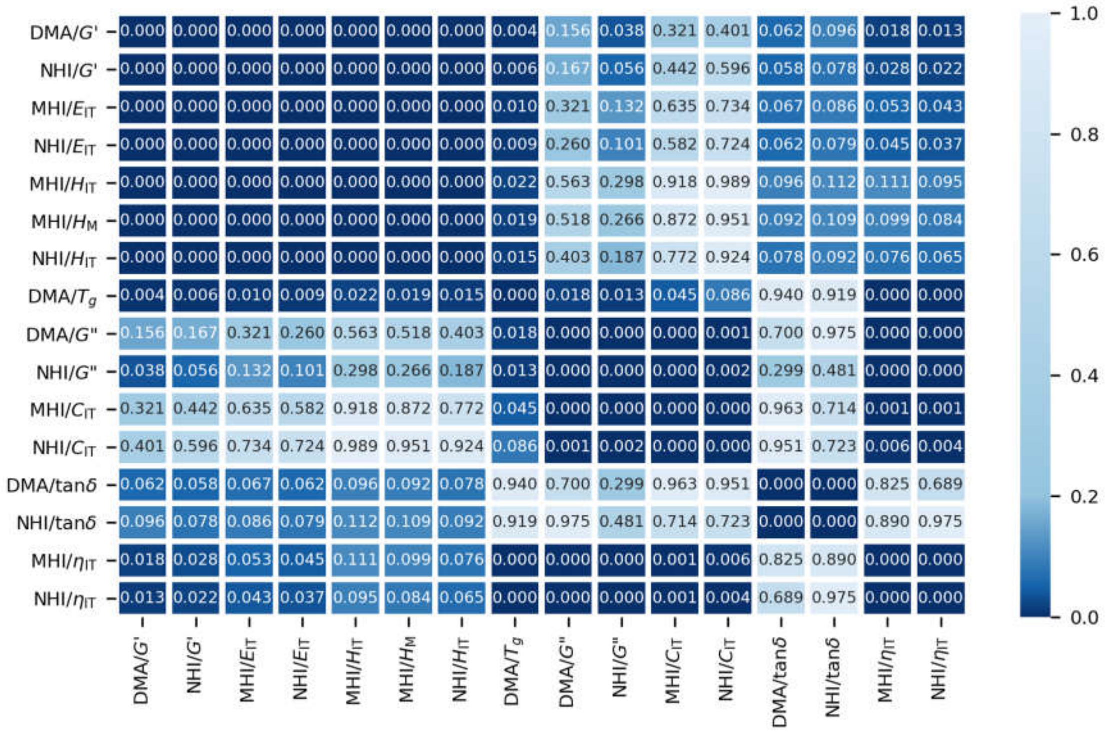

Figure S4. Correlation matrix table showing *p*-values for all pairs of experimentally determined properties in macro-, micro- and nanoscale. The properties in the table are: storage modulus, loss modulus, loss factor and glass transition temperature from dynamic mechanical analysis (DMA/*G*', DMA/*G*", DMA/tan(δ) and DMA/T_g), analogous properties from dynamic nanoindentation experiments (NHI/*G*', NHI/*G*" and NHI/tan(δ)), indentation hardness, modulus, elastic part of indentation work and creep from quasi-static microindentation experiments (MHI/H_{IT}, MHI/E_{IT}, MHI/η_{IT} and MHI/C_{IT}), and analogous properties from quasi-static nanoindentation experiments (NHI/H_{IT}, NHI/E_{IT}, NHI/η_{IT} and NHI/C_{IT}). The properties are given in specific order so as to emphasize their correlations. The table is presented as a heatmap (more intensive color means stronger correlation).

5 Differences among values of macro-, micro- and nanomechanical properties

Figure S5 shows ratios between corresponding macro-, micro- and nanomechanical properties of all investigated samples. For quasistatic properties (Figure S5a) we calculated four ratios: NHI/E_{IT} : MHI/E_{IT} (blue points), NHI/H_{IT} : MHI/H_{IT} (orange), NHI/C_{IT} : MHI/C_{IT} (green), and NHI/E_{IT} : MHI/E_{IT} (red). For dynamic properties (Figure S5b) we calculated three ratios: NHI/*G*' : DMA/*G*' (blue points), NHI/*G*" : DMA/*G*" (orange), and NHI/tanδ : DMA/tanδ (green). If the mechanical properties at all length scales were the same, all ratios should be equal to 1. In our case, most of the ratios are within interval 0.5–1.5, which means that most of the values do not differ by more than 50 %. This is quite acceptable result. As explained in the main text of the manuscript, the differences among macro-, micro- and nanomechanical properties are comparable or even lower than those reported in the literature.

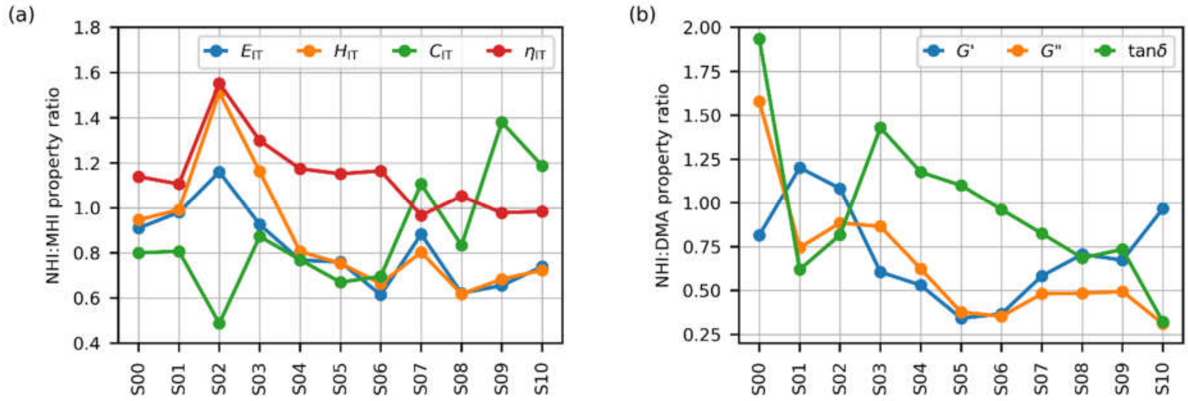

Figure S5. Ratios between corresponding values of mechanical properties from (a) the quasistatic measurements and (b) the dynamic measurements. The ratios of quasi-static properties in the left figure are: NHI/E_{IT} : MHI/E_{IT} (blue), NHI/H_{IT} : MHI/H_{IT} (orange), NHI/C_{IT} : MHI/C_{IT} (green), and NHI/E_{IT} : MHI/E_{IT} (red). The ratios of dynamic properties in the right figure are: NHI/*G*' : DMA/*G*' (blue), NHI/*G*" : DMA/*G*" (orange), and NHI/tanδ : DMA/tanδ (green).

The numerical values of all calculated ratios of all corresponding quasistatic and dynamic properties are summarized in Tables S5 and S6, respectively.

Table S5. Ratios between corresponding quasistatic properties from NHI and MHI.

Sample ID	$\frac{NHI/E_{IT}}{MHI/E_{IT}}$	$\frac{NHI/H_{IT}}{MHI/H_{IT}}$	$\frac{NHI/C_{IT}}{MHI/C_{IT}}$	$\frac{NHI/\eta_{IT}}{MHI/E\eta_{IT}}$
S00	0.91	0.95	0.80	1.14
S01	0.98	0.99	0.81	1.11
S02	1.16	1.51	0.49	1.55
S03	0.93	1.16	0.87	1.30
S04	0.77	0.81	0.77	1.17
S05	0.76	0.75	0.67	1.15
S06	0.61	0.66	0.70	1.17
S07	0.88	0.80	1.11	0.97
S08	0.62	0.62	0.83	1.05
S09	0.66	0.68	1.38	0.98
S10	0.74	0.72	1.19	0.98

Table S6. Ratios between corresponding dynamic properties from NHI and DMA.

SampleID	$\frac{\mathrm{NHI}/\boldsymbol{G}'}{\mathrm{DMA}/\boldsymbol{G}'}$	$\frac{\mathrm{NHI}/\boldsymbol{G}"}{\mathrm{DMA}/\boldsymbol{G}"}$	$\frac{\mathrm{NHI}/\tan\boldsymbol{\delta}}{\mathrm{DMA}/\tan\boldsymbol{\delta}}$
S00	0.82	1.58	1.93
S01	1.20	0.74	0.62
S02	1.08	0.89	0.82
S03	0.61	0.87	1.43
S04	0.53	0.62	1.18
S05	0.34	0.38	1.10
S06	0.37	0.35	0.96
S07	0.58	0.48	0.83
S08	0.71	0.48	0.69
S09	0.67	0.49	0.73
S10	0.97	0.31	0.32

As for quasistatic properties (Table S5), great majority of the ratios oscillated around the ideal value of 1, ranging from 0.6 to 1.2. Slightly higher deviation of S02 sample could be attributed to the fact that the temperature of the measurement was close to the sample T_g (Table 1 in the main text) and thus the final mechanical properties were strongly influenced by even small changes of temperature during measurement (as documented in Fig. 6 in the main text). For sample S09 the slightly higher C_{IT} ratio seemed to be just a small experimental error.

As for dynamic properties (Table S6), most of the ratios was within the interval of 0.5–1.2. For sample S00 the higher ratios could be associated with lower precision of DMA analysis – mechanical properties of very stiff samples are sensitive to the fixing of the specimens. For samples S03–S05 the lower ratios probably resulted from the vicinity of the measurement temperature and T_g, as discussed above. Finally, for sample S10, the slightly lower ratios of *G*” and tanδ could due to lower precision of the experiments as the sample was extremely soft.

6 Definitions and different values of H_{IT} and H_M

Figure S6 summarizes the difference between definition and calculation of indentation hardness (H_{IT}) and Martens hardness (H_M). The figure is valid for microindentation with Vickers indenter (diamond square pyramid with angle between two non-adjacent faces = 136 deg), but the principle is the same also for nanoindentation and/or other indenter geometries. All types of hardness are calculated using the same general formula ($H = F/A$, where $F = F_{max}$ = maximum loading force and A = area of the imprint on the polymer surface), but differences arise concerning definition of the A. The indentation hardness is defined by means of *projected area* ($A = A_p$; Figure S6a) and calculated in terms of Oliver & Pharr theory, which assumes *sink-in* effect that leads to further decrease in projected area (bottom of Figure S5a). Martens hardness (also known as universal hardness) is defined by means of *developed area* ($A = A_d$; Figure S6b) and calculated directly from the experimental value of h_M, without additional theoretical assumptions (bottom of Figure S5b).

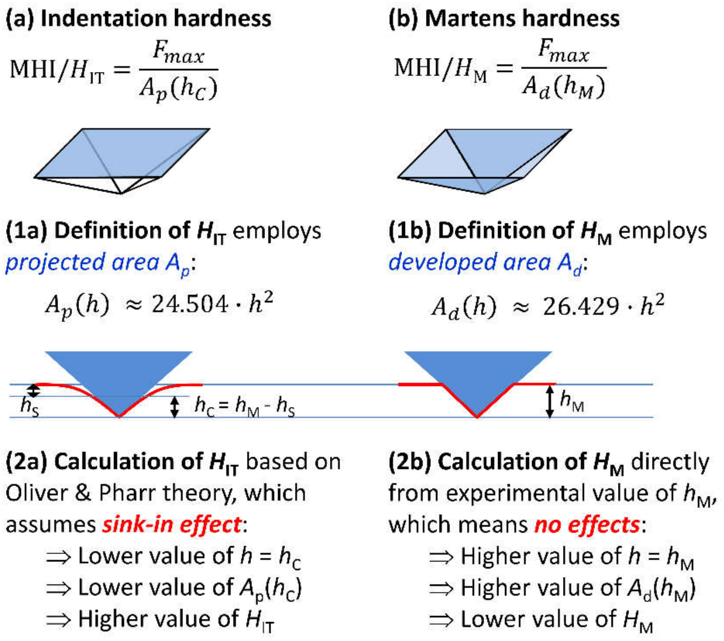

Figure S6. Differences between definition and calculation of indentation hardness (a), and Martens hardness (b). The scheme is valid for microindentation with Vickers indenter (diamond square pyramid with angle between two non-adjacent faces = 136 deg). The symbols h_M, h_S, and h_C represent maximum penetration depth, penetration depth associated with sink-in effect and contact penetration depth (calculated as h_M-h_S), respectively.

The values of H_{IT} are expected to be higher than the values of H_M just due to the fact that $A_p < A_d$. For Vickers indenter we get:

$$\frac{H_{IT}}{H_M} = \frac{F_{max}/A_p(h)}{F_{max}/A_d(h)} = \frac{A_d(h)}{A_p(h)} \approx \frac{26.429h^2}{25.504h^2} \approx 1.079 \qquad \text{(S1)}$$

Moreover, the H_{IT}/H_M ratio is further increased due to the different evaluation of the two quantities. The value of H_{IT} is corrected for sink-in effect (assumption of Oliver & Pharr theory; ref. [Oliver 1992; Oliver 2010]), which further decreases the value of A_p and, consequently increases the value of $H_{IT} = F_{max}/A_p$.

References

[Balta-Calleja 2000a] Balta-Calleja, F.J.; Fakirov, S: Microhardness of Polymers; Cambridge University Press: Cambridge, UK, 2000; doi:10.1017/CBO9780511565021.

[Balta-Calleja 2004] Balta-Calleja F.J.; Flores, A.; Michler, G.H. Microindentation Studies at the Near Surface of Glassy Polymers: Influence of Molecular Weight. *J. Appl. Polym. Sci.* **2004**, *93*, 1951–1956; doi: 10.1002/app.20665.

[Fakirov 1999] Fakirov, A.; Balta-Calleja, F.J.; Krumova, M. On the Relationship between Microhardness and Glass Transition Temperature of Some Amorphous Polymers. *J. Polym. Sci. B Polym. Phys.* **1999**, *37*, 1413–1419; doi: 10.1002/(SICI)1099-0488(19990701)37:13<1413::AID-POLB7>3.0.CO;2-Q.

[Fakirov 2000] Fakirov, S.; Krumova, M.; Krasteva, B. On the temperature dependence of microhardness of some glassy polymers. *J. Mater. Sci. Lett.* **2000**, *19*, 2123–2125; doi: 10.1023/A:1026762408839.

[Flores 2009] Flores, A.; Ania, F.; Balta-Calleja, F.J. From the glassy state to ordered polymer structures: a microhardness study. *Polymer* **2009**, *50*, 729–746; doi: 10.1016/j.polymer.2008.11.037.

[Gedde 1995] Gedde, U.W. *Polymer Physics*; Chapman & Hall: London, UK, 1995.

[Kupka 2019] Kupka, V.; Zhou, Q.; Ansari, F.; Tang, H.; Slouf, M.; Vojtova, L.; Berglund, L.A.; Jancar, J. Well-dispersed polyurethane/cellulose nanocrystal nanocomposites synthesized by a solvent-free procedure in bulk. *Polym. Compos.* **2019**, *40*, E456–E465; doi:10.1002/pc.24748.

[Oliver 1992] Oliver, W.C.; Pharr, G.M. An improved technique for determining hardness and elastic modulus using load and displacement sensing indentation experiments. *J. Mater. Res.* **1992**, *7* 1564–1583; doi: 10.1557/JMR.1992.1564.

[Oliver 2010] Oliver, W.C., Pharr, M.G. Nanoindentation in Materials Research: Past, present, and future. *MRS Bull.* **2010**, *35*, 897–907; doi:10.1557/mrs2010.717.

[Ostafinska 2015] Ostafinska, A.; Fortelny, I.; Nevoralova, M.; Hodan, J.; Kredatusova, J.; Slouf, M. Synergistic effects in mechanical properties of PLA/PCL blends with optimized composition, processing, and morphology. *RSC Adv.* **2015**, *5*, 98971–98982; doi: 10.1039/C5RA21178F.

[Ramsdale-Capper 2018] Ramsdale-Capper, R.; Foreman, J.P. Internal antiplasticisation in highly crosslinked amine cured multifunctional epoxy resins. *Polymer* **2018**, *146*, 321–330; doi: 10.1016/j.polymer.2018.05.048.

[Rishina 2019] Rishina, L.A.; Kissin, Y.V.; Lalayan, S.S.; Krasheninnikov, V.G. Synthesis of atactic polypropylene: Propylene polymerization reactions with $TiCl_4$–$Al(C_2H_5)_2Cl/Mg(C_4H_9)_2$ catalyst. *J. Appl. Polym. Sci.* **2019**, *136*, 47692; doi: 10.1002/app.47692.

[Slouf 2018b] Slouf, M.; Pilar, J.; Dybal, J.; Sloufova, I.; Michalkova, D.; Lukesova, M.; Zgadzai, O.; Blank, A.; Filippov, S.K. UV degradation of styrene-butadiene rubber versus high density poly(ethylene) in marine conditions studied by infrared spectroscopy, micro indentation, and electron spin resonance imaging. *Polym. Degrad. Stab.* **2018**, *156*, 132–143; doi: 10.1016/j.polymdegradstab.2018.08.005.

[Tan 1999] Tan, N.C.B.; Bauer, B.J.; Plestil J.; Barnes, J.D.; Liu, D.; Matejka, L.; Dusek, K.; Wu, W.L. Network structure of bimodal epoxies—a small angle X-ray scattering study. Polymer 40 (1999) 4603–4614. DOI: https://doi.org/10.1016/S0032-3861(99)00096-8.

[Ward 2004] Ward, I.M.; Sweeney, J. *An Introduction to the Mechanical Properties of Solid Polymers*, 2nd ed.; John Wiley & Sons: Chichester, UK, 2004.

[Wu 1986a] Wu, W.L.; Bauer B.J. Network structure of epoxies — a neutron scattering study: 2. Polymer 27 (1986) 169-180. DOI: https://doi.org/10.1016/0032-3861(86)90322-8.

[Wu 1986b] Wu, W.L.; Bauer, B.J. Epoxy network structure. 3. Neutron-scattering study of epoxies containing monomers of different molecular weight. Macromolecules 19 (1986) 1613-1618. DOI: https://doi.org/10.1021/ma00160a021.

[Yin 2013] Yin, H.; Jin, H.; Wang, C.; Sun, Y.; Yuan, Z.; Xie, H.; Wang, Z.; Cheng, R. Thermal, damping, and mechanical properties of thermosetting epoxy-modified asphalts. *J. Therm. Anal. Calorim.* **2014**, *115*, 1073–1080; doi: 10.1007/s10973-013-3449-9.
